# Genetic and Antigenic Evolution of European Swine Influenza A Viruses of HA-1C (Avian-Like) and HA-1B (Human-Like) Lineages in France from 2000 to 2018

**DOI:** 10.3390/v12111304

**Published:** 2020-11-13

**Authors:** Amélie Chastagner, Séverine Hervé, Stéphane Quéguiner, Edouard Hirchaud, Pierrick Lucas, Stéphane Gorin, Véronique Béven, Nicolas Barbier, Céline Deblanc, Yannick Blanchard, Gaëlle Simon

**Affiliations:** 1 Swine Virology Immunology Unit, Ploufragan-Plouzané-Niort Laboratory, ANSES, BP53, 22440 Ploufragan, France; amelie.chastagner@anses.fr (A.C.); severine.herve@anses.fr (S.H.); stephane.queguiner@anses.fr (S.Q.); stephane.gorin@anses.fr (S.G.); nicolas.barbier@anses.fr (N.B.); celine.deblanc@anses.fr (C.D.); 2 Viral Genetic and Biosecurity Unit, Ploufragan-Plouzané-Niort Laboratory, ANSES, BP53, 22440 Ploufragan, France; edouard.hirchaud@anses.fr (E.H.); pierrick.lucas@anses.fr (P.L.); veronique.beven@anses.fr (V.B.); yannick.blanchard@anses.fr (Y.B.)

**Keywords:** swine influenza, virus evolution, genetic diversity, antigenic drift, H1N1, H1N2, genotype, matrix protein, surveillance, Eurasian avian-like lineage

## Abstract

This study evaluated the genetic and antigenic evolution of swine influenza A viruses (swIAV) of the two main enzootic H1 lineages, i.e., HA-1C (H1_av_) and -1B (H1_hu_), circulating in France between 2000 and 2018. SwIAV RNAs extracted from 1220 swine nasal swabs were hemagglutinin/neuraminidase (HA/NA) subtyped by RT-qPCRs, and 293 virus isolates were sequenced. In addition, 146 H1_av_Ny and 105 H1_hu_Ny strains were submitted to hemagglutination inhibition tests. H1_av_N1 (66.5%) and H1_hu_N2 (25.4%) subtypes were predominant. Most H1 strains belonged to HA-1C.2.1 or -1B.1.2.3 clades, but HA-1C.2, -1C.2.2, -1C.2.3, -1B.1.1, and -1B.1.2.1 clades were also detected sporadically. Within HA-1B.1.2.3 clade, a group of strains named “Δ146-147” harbored several amino acid mutations and a double deletion in HA, that led to a marked antigenic drift. Phylogenetic analyses revealed that internal segments belonged mainly to the “Eurasian avian-like lineage”, with two distinct genogroups for the M segment. In total, 17 distinct genotypes were identified within the study period. Reassortments of H1_av_/H1_hu_ strains with H1N1pdm virus were rarely evidenced until 2018. Analysis of amino acid sequences predicted a variability in length of PB1-F2 and PA-X proteins and identified the appearance of several mutations in PB1, PB1-F2, PA, NP and NS1 proteins that could be linked to virulence, while markers for antiviral resistance were identified in N1 and N2. Altogether, diversity and evolution of swIAV recall the importance of disrupting the spreading of swIAV within and between pig herds, as well as IAV inter-species transmissions.

## 1. Introduction

Influenza A viruses (IAVs) are members of the *Orthomyxoviridae* family and are composed of eight negative single-stranded RNA segments. The lack of RNA polymerase proofreading activity during virus replication produces a continuous diversity of variants and the segmented genome allows gene reassortment between virus strains in case of co-infection [1]. Inter-species transmissions also play an important role in IAV evolution. They provide new sources of diversity, either following adaptation to a new host of a virus transmitted in toto, or through reassortment between a virus infecting another species and a virus strain already adapted in this species [2]. Amino acid mutations or segment exchanges can alter the virulence and pathogenicity, the antiviral resistance, escape to host immunity or vaccine protection, or further help to jump host [3]. When they occur in, or concern, the surface glycoproteins, i.e., the hemagglutinin (HA) and/or neuraminidase (NA) encoding genes, genetic modifications and reassortments can result in antigenic drift and shift, respectively [4]. Competition between strains, environmental conditions and host immune system create selective pressures, which limit the diversity of IAV within each host [5,6]. Thus, many new variants or reassortant viruses may be only sporadically detected, but some of them may also expend in naïve immune populations.

IAVs are classified into HxNy subtypes defined by the nature of HA and NA glycoproteins. In numerous host species, several genetic lineages are then distinguished within each subtype [7]. Thus, four lineages of swine IAVs (swIAVs) are currently enzootic in the pig population in Europe: (i) the “avian-like swine H1N1” (H1_av_N1; clade HA-1C) lineage that emerged in 1979 following transmission of a H1N1 virus from duck to pig and in toto adaptation [8]; (ii) the “human-like reassortant swine H3N2” (H3N2) lineage that emerged in 1984 after a reassortment between a human-like swine H3N2 (A/Port Chalmers/1/73-like virus) and a H1_av_N1 strain [9]; (iii) the “human-like reassortant swine H1N2” (H1_hu_N2; clade HA-1B) lineage that emerged in 1994, resulting from a reassortment between the swine H3N2 and a seasonal human H1N1 [10]; (iv) the “pandemic-like swine H1N1” (H1N1pdm; clade HA-1A.3.3.2) which was transmitted from humans and quickly spread in pig populations worldwide after the pandemic of 2009 [11,12]. The co-circulation of swIAVs from these four lineages in the pig population allows the emergence of reassortant viruses combining genes from these different enzootic swIAVs [13]. Such reassortant viruses are detected sporadically but some of them have adapted locally. Thus, the diversity of swIAVs has changed in several European countries since 2000 following the setup of a new H1_av_N2 reassortant in Denmark in 2003 which has replaced the European H1_hu_N2 [14], and especially since 2009 with the emergence of numerous reassortant viruses containing one or more gene(s) from the H1N1pdm. In the United Kingdom, the H1N1pdm virus and a reassortant H1_hu_N2 virus with internal segments originating from the H1N1pdm lineage has replaced almost completely the previous H1_av_N1 and H1_hu_N2 viruses [13]. In Denmark, the H1N1pdm and the local H1_av_N2 viruses were also more frequently detected than the H1_av_N1 virus in recent years [13,15]. In Germany and Italy, the frequency of reassortant viruses harboring at least one H1N1pdm segment has increased continuously from 2009 [16,17,18,19,20,21].

In France, swIAV is a major agent responsible for acute respiratory syndromes in pig herds [22]. The French pig production system is predominated by farrow-to-finish farms (50.4%), followed by growing (29.8%) and post-weaners-to-finish farms (13.5%), with a mean farm size of 1200 pigs [23]. All types of farm, either with or without breeders, are concerned by swIAV infections, but growing pigs of all ages are more frequently detected as infected than gilts and sows [22]. The surveillance reports drawn up before 2013 reported a dominance of H1_av_N1 and H1_hu_N2 viruses, along with sporadic detections of reassortant viruses between these two enzootic lineages, whereas the H3N2 was rarely detected [12,13,24]. The H1N1pdm virus was detected as early as 2010 but its diffusion in the territory seemed much slower than in other countries. Its spread and evolution until 2017 was specifically described previously [25]. The aim of the present study was to better characterize and describe the genetic and antigenic evolution of H1_av_Ny and H1_hu_Ny viruses detected in pigs in France from 2000 to 2018. This work should help to identify strains harboring virulence markers and to provide accurate information to keep detection tools and the vaccine strains updated.

## 2. Materials and Methods

### 2.1. Biological Samples, swIAV Detection and Preliminary Molecular Subtyping

Nasal swabs (MW950Sent2mL Virocult^®^, Kitvia, Labarthe-Inard, France) were gathered by the French National Reference Laboratory (NRL) for Swine Influenza, Ploufragan, France. They were collected from 2005 to 2018 from pigs with acute respiratory disease, in the context of veterinary requests for IAV diagnostic analyses, dedicated epidemiological investigations conducted by the French Agency for Food, Environmental and Occupational Health & Safety (ANSES, Ploufragan-Plouzané-Niort Laboratory, Ploufragan, France), or the passive surveillance program implemented by the national surveillance network for swIAVs in pigs (Résavip). Nasal swab supernatants were first screened for detection of IAV genome with conventional or real-time RT-PCR assay targeting the matrix (M) gene, using commercial or in-house methods [26]. Positive RNA extracts were then subtyped with in-house real-time RT-PCR assays developed for specific identifications of the HA and NA genetic lineages (H1_av_, H1_hu_, H1pdm, H3, N1, N1pdm and N2) of European swIAVs [26,27]. All samples in which several HA and/or NA subtypes were detected (virus mixtures) were excluded from the study.

### 2.2. Virus Isolation

All M-gene positive samples with Ct < 30 were selected for virus isolation (only one sample per farm at a given sampling date), except for supernumerary H1_av_N1 viruses from the Brittany region for which we selected a representative panel based on location and period of detection. Selected M-gene positive samples were subjected to virus propagation by one passage onto 9-day-old specific pathogen-free (SPF) embryonated chicken eggs or Madin-Darby Canine Kidney (MDCK) cells, according to standard procedures [28]. For swine antisera production and in some cases for antigenic characterization, reference virus strains and/or swine H1Ny isolates first isolated on MDCK cells were further propagated by a second passage on eggs or MDCK cells. Harvested cell culture supernatants and allantoic fluids were clarified by centrifugation and stored at −70 °C until sequencing, pig inoculation, or hemagglutination inhibition assays.

Additional virus strains propagated on embryonated chicken eggs from biological samples, all taken in 2000–2004, were kindly provided by LABOCEA 22, Ploufragan. These allantoic fluids were submitted to molecular subtyping, as described above, before sequencing.

### 2.3. Sequencing

Viral RNA was extracted from viral cultures using the NucleoSpin^®^ RNA Kit (Macherey-Nagel, Düren, Germany). The nucleotide sequences were obtained either by the Sanger method on an Applied Biosystems^®^ Sanger Sequencing 3500 Series Genetic Analyzer (Hitachi High Technologies, Naka, Japan) or by next-generation sequencing (NGS) on Thermo Fisher Scientific’s Ion Proton instrument (Thermo Fisher, Carlsbad, CA, USA). The reads obtained by NGS were cleaned with Trimmomatic 0.36 software [29], then assembled by mapping on reference genomes using Burrows-Wheeler Aligner software (version 0.7.15-r1140 [30]) and by de novo using the SPAdes and MIRA programs (versions v3.10.0 [31] and 4.0.2 [32]). A single consensus sequence per viral segment was generated by combining the contigs produced using both methods with Vector NTI Advance 11.0 software (Invitrogen Corp., Carlsbad, CA, USA). Sequences obtained by NGS for this study were available in GenBank: Bioproject # PRJNA623701. Genbank identification numbers of the sequences used in this study are reported in Supplementary Table S1.

### 2.4. Phylogenetic Analyses of HA-, NA- and M-Encoding Genes

The sequences obtained in this study, and sequences of other swIAVs isolated in France and previously deposited in public databases, were compared to swIAV sequences retrieved from the Influenza Research Database (IRD) (available at https://www.fludb.org/). Phylogenetic trees were generated by a Bayesian approach using BEAST v1.8.4 [33], with the substitution models ‘HKY + Gamma + Codon Position (CP)’ for HA- and NA-genes or ‘GTR + Gamma + CP’ for M gene, improved by the sample collection years [34]. A Markov chain Monte Carlo (MCMC) method of 10,000,000 samplings for every 1000 was performed. The quality of the posterior distribution of each setting was checked through the Effective Sample Size (ESS) with Tracer v1.6 [33], with 10% of burning. A minimum ESS value of 150 was accepted for prior when the values were over 300 for posterior, likelihood and clock-rate settings. Trees were summarized applying 10% of burning with TreeAnnotator v1.8.4 [33] and visualized with FigTree v1.4.3 [35]. The HA clades were defined according to the global swine H1 clade scheme [7], using the ‘Swine H1 Clade Classification Tool’ accessible in IRD.

### 2.5. Large-Scale Phylogeny of M-Encoding Segment

A large-scale phylogenetic analysis was performed to evaluate the origin of the divergent phylogenetic clusters identified among M gene sequences from French swIAVs. After checking that the M gene sequences obtained in this study formed a distinct cluster from M genes of avian IAV lineages as previously described [36], we focused the large-scale analysis on all sequences of M segments from swIAV isolated in the Northern hemisphere, and human IAV isolated in Europe when associated with a known HA/NA subtype (sequences available in IRD at the 16th of November 2017). In total 12,386 complete M gene sequences were selected after cleaning, representing 7154 M-gene sequences of swIAVs from the Northern hemisphere and 5232 European human IAV strains. The sequences were aligned with MAFFT v7.311 [37], then a cluster analysis was performed with R 3.4.0 using the packages ‘phyclust’ and ‘ape’. The phylogenetic clustering was defined by the main function ‘phyclust’, which uses an evolutionary Continuous Time Markov Chain model-based approach to identify population structure from DNA data.

### 2.6. Amino Acid Sequence Analyses

DNA sequences were translated into amino acid sequences with Seaview 4.6.1 [38]. For all segments, amino acid positions were numbered from the first methionine. Thus, the amino acid numbering included the signal peptide for HA. The variability of peptides in protein sequence alignments was measured by the entropy values with the Antigen Variability ANAlyzer 0.31 (AVANA) software with default parameters [39]. Genogroup-specific amino acid mutations were identified using two methods: (i) the genogroup pairwise comparison in AVANA to identify all genogroup-specific mutations without a priori; (ii) the R 3.4.0 analysis only of residues identified to be involved in change of pathogenicity, virulence or fitness of IAVs, according to the literature (Table S2). Antigenic and receptor-binding sites in HA of H1 viruses were determined according to the literature [40,41], as well as those of NA which were deduced from descriptions of N2 viruses [42,43].

### 2.7. Antigenic Characterization

Swine antisera against swIAV strains representative of European enzootic lineages of H1 subtype and/or H1 swIAVs isolated in France during the study period were produced in SPF pigs at ANSES facilities (see [25] for details) (Table 1). Strain A/Sw/England/117316/86, a representative of the formal “classical swine H1N1” (clswH1N1) lineage which was transiently isolated in Europe in the 1980s, was also included, as it belongs to the HA-1A clade as H1N1pdm strain.

Hemagglutination inhibition (HI) assays were performed according to standard procedures [28]. Briefly, four hemagglutinating units (HAU) of egg-propagated virus were incubated with two-fold dilutions (starting at dilution 1:10) of swine antiserum and tested against 0.5% chicken red blood cells. HI antibody titers were expressed as the reciprocal of the highest dilution inhibiting 4 HAU of virus. The means of HI antibody titers obtained by groups of strains were compared with R 3.4.0 using the function ‘pairwise.wilcox.test’ on log-transformed HI titers without adjustment. Difference of means was significant for *p* < 0.05. The H1 antibody titers <10 were replaced by 1 within the test to give no weight in the mean of log-transformed value.

## 3. Results

### 3.1. Relative Proportions of H1_av_Ny and H1_hu_Ny swIAVs in France between 2000 and 2018

**Figure 1:**
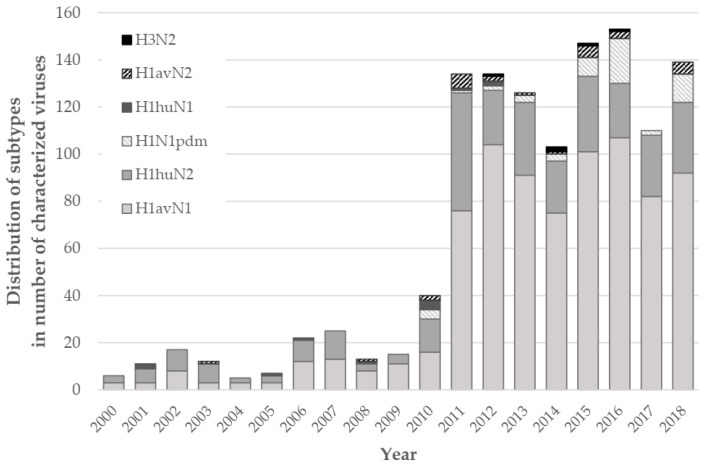
Yearly distribution of swine influenza A viruses (swIAV) identified in France from 2000 to 2018. Data based on molecular subtyping using European swIAV hemagglutinin- (HA)- and neuraminidase- (NA)-specific RT-PCRs.

In this study, 1220 swIAVs from as many independent respiratory outbreaks that occurred from 2000 to 2018 in pigs reared in mainland France were HA/NA subtyped molecularly. From 2000 to 2009, less than 25 virus strains were subtyped each year (N = 133). The HA- and NA-specific RT-PCRs allowed discriminating 67 H1_av_N1 strains (50.4%), 59 H1_hu_N2 strains (44.4%), five reassortant H1_hu_N1 strains (3.8%) and two reassortant H1_av_N2 strains (1.5%), but no H3N2 strain. From 2010, the yearly number of subtyped viruses increased more than five-fold thanks to the establishment of the national swIAV surveillance network. In total, 1087 swIAVs were subtyped between 2010 and 2018: 744 H1_av_N1 strains (68.4%), 251 H1_hu_N2 strains (23.1%), 54 H1N1pdm strains (5.0%), 25 reassortant H1_av_N2 strains (2.3%), seven reassortant H1_hu_N1 strains (0.6%), five H3N2 strains (0.5%) and one reassortant H1pdmN1 (0.1%). No reassortant of H3N1 subtype, or reassortant exhibiting the H1_av_- or H1_hu_-encoding gene with the N1pdm segment were detected. Thus, viruses with an HA belonging either to the H1_av_ or the H1_hu_ lineages represented more than 95% of the swIAVs identified from 2000 to 2018. Despite H1N1pdm introduction in 2010 [25], H1_av_N1 swIAVs have remained the predominant viruses since 2004, with an annual frequency varying from 40% to 77%, while H1_hu_N2 viruses counted for 15% to 48% (Figure 1).

### 3.2. Genetic and Antigenic Evolution of HA-1C (H1_av_Ny) swIAVs Isolated in France

**Figure 2:**
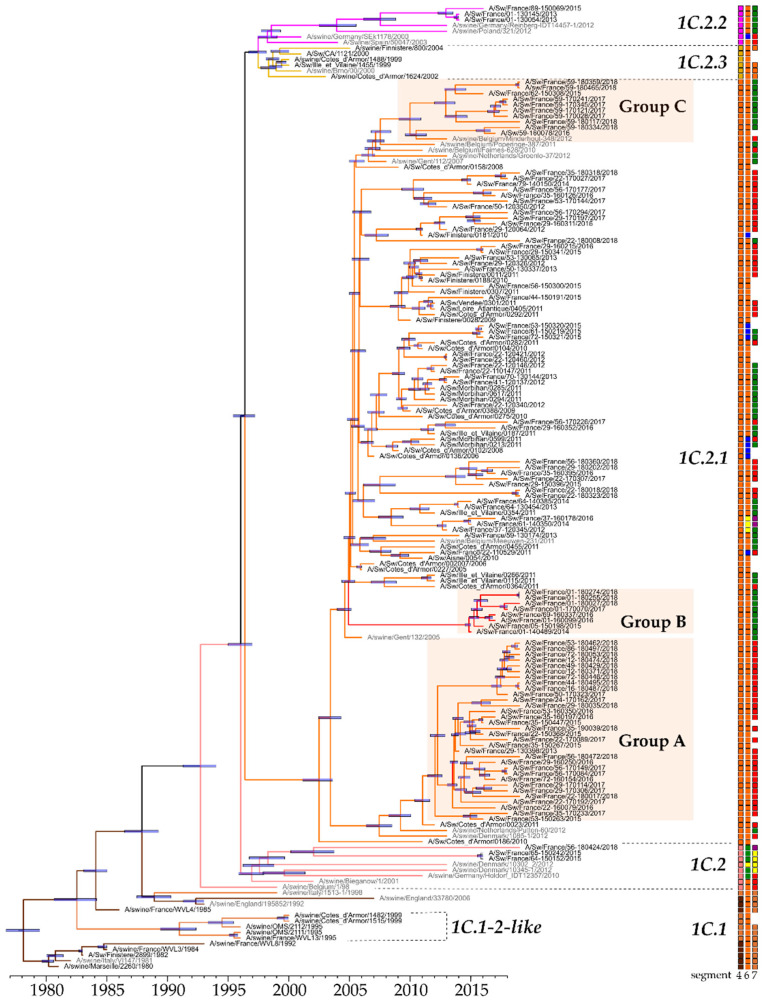
Bayesian inference tree of HA-1C (H1_av_N_y_) swIAV strains. Strains isolated in pigs in France are indicated in black type whereas those isolated in pigs in other countries are in grey type. Nodes supported by more than 50% of the sampled trees are indicated by a blue bar displaying the 95% credibility intervals of highest posterior density for the node heights. HA-1C clades are defined according to the classification of Anderson et al., 2016 [7]. The three boxes on the right grid are colored according to the genogroups defined on genomic segments 4 (HA), 6 (NA) and 7 (M) phylogenies, respectively. For segment 6, the boxes were colored in orange for N1 ‘Eurasian avian-like’ lineage, in blue for N2 ‘Scotland-like’ lineage, in yellow for N2 of ‘H3N2 human-like’ lineage, and in green for N2 of ‘Gent-like’ lineage (see Section 3.4.1). For segment 7, the boxes were colored in brown for ‘M ancestral Eurasian avian-like swine’ group, in red for ‘M recent Eurasian avian-like swine’ group, in green for ‘M European swine’ group, in yellow for ‘M Danish-like’ group, and in purple for M segment from the H1N1pdm lineage (see Section 3.4.2).

Between 2000 and 2018, 182 out of 838 H1_av_Ny strains were sequenced. One hundred and thirty four of these (116 H1_av_N1 and 18 H1_av_N2), for which at least both HA- and NA-encoding genes were completely sequenced, were included in phylogenetic analyses. Phylogenetic analyses revealed that the HA-encoding genes of H1_av_Ny strains isolated in France since 1980 classified into different clades (Figure 2). According to the swIAV H1 classification [7], the swIAV sequences from the oldest virus strains isolated in France and retrieved from public databases, i.e., A/Swine/Marseille/2260/1980(H1_av_N1) and A/Swine/Finistere/2899/1982(H1_av_N1), belonged to clade 1C.1, within the 1C “Eurasian avian lineage”. From 1980 to 2000, strains from this clade, as well as from 1C.1-2-like clade derived from the former, were predominant in France, as elsewhere in Europe. Between 1999 and 2004, strains closed to A/Swine/Ille-et-Vilaine/1455/1999(H1_av_N1) and belonging to clade 1C.2.3 progressively replaced strains from the previous clades 1C.1 and 1C.1-2-like, before being supplanted themselves by strains belonging to clade 1C.2.1, predominant from 2005 to 2018. Conversely, few strains identified between 2013 and 2018 grouped in clade 1C.2 which is preferentially located in Denmark and Northern Europe, or in clade 1C.2.2 which is mostly encountered in Western and Southern Europe.

**Figure 3:**
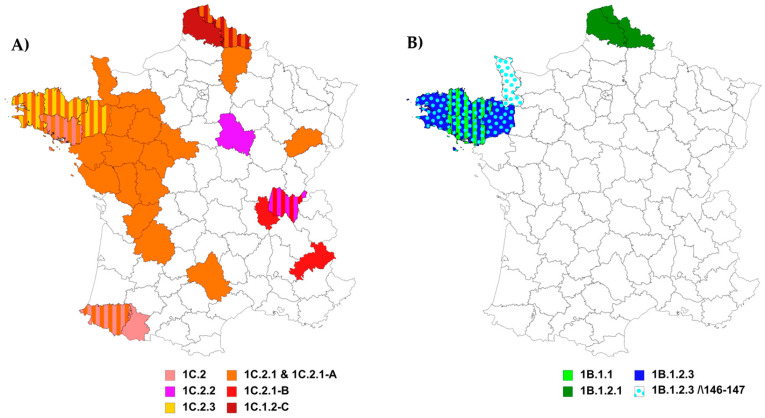
Geographical distribution of swIAVs of HA-1C/H1_av_ (**A**) and HA-1B/H1_hu_ (**B**) lineages identified from 2000 to 2018 in mainland France. The grey lines define the limits of each French administrative “département”. A “département” is colored according to the H1 clade when at least one strain of the clade was identified in this area. Stripes are used when several genogroups were identified in the same “département”.

Within the HA-1C.2.1 clade, three genogroups named A, B and C, respectively, can be pointed out, based on large numbers of substitutions on non-silent sites compared with A/Sw/Cotes d’Armor/0388/09 (CA0388/09), a representative strain for clade 1C.2.1, and/or geographical locations. Group A gathered recent strains, isolated from 2013 to 2018, from all over the territory. This group presented specific mutations in HA, such as A11T, Q68H, N101K, A113T, T149S, S154T, A158V, G172A, L178I, Q225K, Q253K (in 9/34 strains), H270Y, N279S, H288N, E516K and V545I, compared to CA0388/09. Among these mutations, six were located in antigenic sites of the HA1 subunit, i.e., T149S, S154T, A158V, G172A, L178I, and Q253K. Group B was composed of strains isolated between 2014 and 2018 mainly in the Eastern part of France (Figure 2 and Figure 3A). These strains also harbored several mutations in HA protein as compared to CA0388/09: V14T, N52T, G70E, V74I, K103R, A106T, N142Q, S156L, A158V, S220T, R226K, A241T, Q253R, N275D, and V399I. Five of them, i.e., N142Q, S156L, A158V, S220T and Q253R, were located in antigenic sites in HA1 whereas mutation HA-A241T was in the receptor-binding site (RBS). Finally, group C was composed of strains isolated between 2015 and 2018 in the North of France exclusively (Figure 2 and Figure 3A). These strains harbored many amino acid mutations in HA protein, i.e., L37M, L61M, V74I, N91K, D114E, A158V, L178I, V216I, V232A, N275D/G, M283I, T293S, S305N, K467R, S468L, E508A and I550V as compared to CA0388/09. Among these mutations, HA-N91K and HA-L178I were located in antigenic sites in HA1.

Strains harboring a HA-encoding gene classified into clade 1C.2 were detected in two farms located in the Southwest in 2015 and in one farm in the Northwest in 2018 (Figure 3A). As compared to CA0388/09, Southwestern strains harbored two amino acid deletions in HA1-encoding sequence in position 137 and 147, compared to positions 146 and 172 in the Northwestern one. These three strains also presented many mutations in antigenic sites: L88I, PNH->LSY at positions 141-143, H155N, G172D, N173S, K180Q, T183K, K186R, D202Y and G219V for the Southwestern strains; P141E, V151G, A152S, H155R, A158T, N159K, L178I, N185D, K186R, D202Y, I207A, N211S, G219V and S220T for the Northwestern strain.

Finally, three strains with H1 from clade 1C.2.2 were identified in the Eastern part of France (Figure 3A). They harbored 22 mutations in HA (data not shown), including S179I in an antigenic site.

The antigenic relationships of 146 H1_av_Ny viruses, for which the HA1 sequences were obtained, were tested in HI assays using a selection of swine antisera produced against virus strains representative of the different HA-1C clades encountered in France over time. Hyperimmune serum (HIS) containing antibodies directed against strain Fin2899/82 (clade HA-1C.1) (HIS-Fin2899/82) exhibited slightly weaker mean HI antibody titers than HIS-Mo0070/05 and HIS-CA0388/09 (clade HA-1C.2.1) when tested against H1_av_Ny strains from clade HA-1C.2.1 isolated between 2000 and 2010 (Table 2). Reactions of HIS-Mo0070/05 and HIS-CA0388/09 were significantly higher against strains of clade HA-1C.2.1 isolated in 2011–2014, but lower against strains isolated in a more recent period (2015–2018) (Table 2). It could be noted that the serum produced against a H1_av_N2 reassortant virus of the same HA clade, i.e., HIS-CA0186/10, showed HI titers significantly weaker against strains of clade HA-1C.2.1 than HIS-CA0388/09 and HIS-Mo0070/05 (Table 2). The relationships between the HI antibody titers obtained by HIS-CA0388/09 against each strain of clade HA-1C.2.1 and the observed mutations in antigenic sites or RBS of the tested strains were analyzed with a generalized linear model. This analysis revealed a statistical link between the decrease in HI antibody titers and the appearance of mutation N142D or K180N in HA-1C.2.1 strains. Their prevalence increased from 2014, with an individual estimated effect of -1.8 log for HA-N142D and -1.5 log for HA-K180N on HI antibody titer of HIS-CA0388/09. Interestingly, HIS-CA0388/09 exhibited higher mean HI titers against strains from groups A and C (427.15 and 470.32, respectively) as compared to strains from group B or other strains belonging to clade HA-1C.2.1 and isolated between 2015 and 2018 (246.75 and 265.52, respectively) (Table 2). Although strains from group B harbored mutations in antigenic sites, the HI antibody titers of HIS-CA0388/09 remained similar to those against HA-1C.2.1 parental strains named ‘others’ in Table 2. HIS-65-150242 (H1_av_N2, clade HA-1C.2), but not HIS-CA0186/10 (H1_av_N2, clade HA-1C.2.1), inhibited the hemagglutination properties of three H1_av_N2 strains from clade HA-1C.2 (Table 2). Finally, HIS containing antibodies directed against H1N1pdm (clade HA-1A.3.3.2) and H1_hu_N2 (clade HA-1B.1) virus strains showed no or low HI antibody titers against H1_av_Ny strains, whatever the HA-1C clade they belong to (Table S3A).

### 3.3. Genetic and Antigenic Evolution of HA-1B (H1_hu_Ny) swIAVs Isolated in France

Between 2000 and 2018, 111 out 324 H1_hu_Ny strains were sequenced. Eighty-seven of them (78 H1_hu_N2 and 9 H1_hu_N1), for which at least HA- and NA-encoding genes were completely sequenced, were included in the phylogenetic analyses.

**Figure 4:**
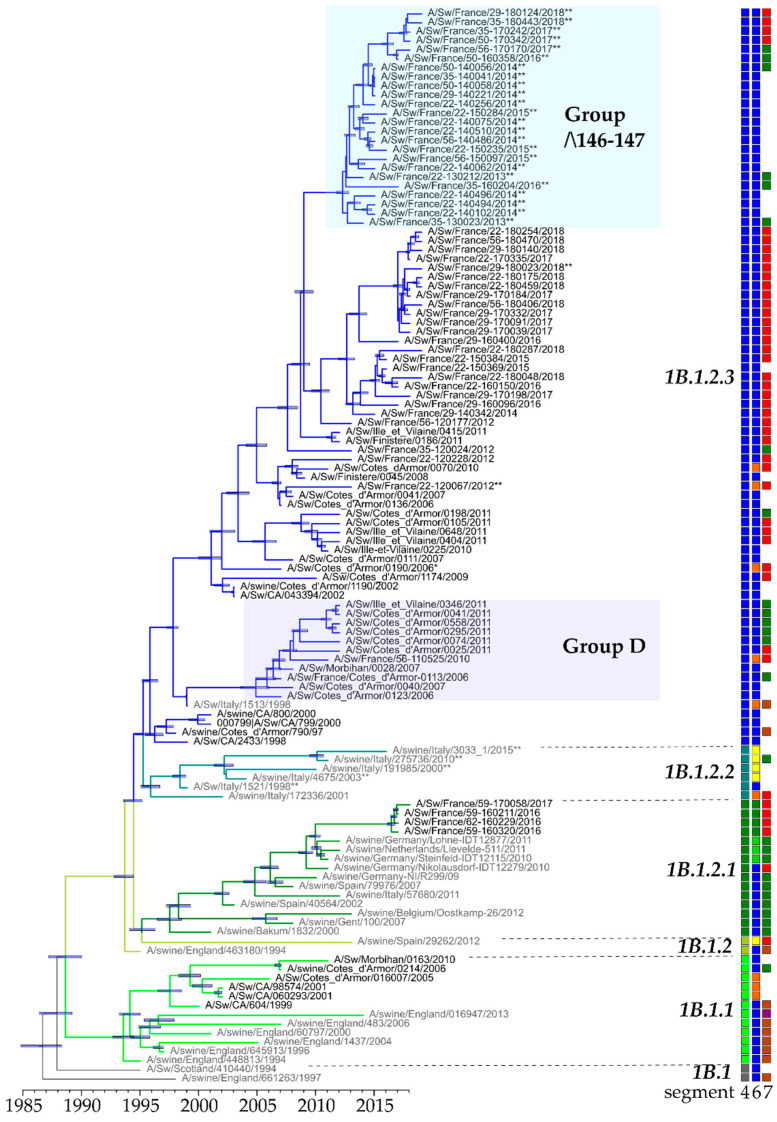
Bayesian inference tree of HA-1B (H1_hu_Ny) swIAV strains. Strains isolated in pigs in France are indicated in black type whereas those isolated in pigs in other countries are in grey type. Strains harboring nucleotide deletions leading to lack of one (*) or two (**) amino acid(s) in positions 147 or 146-147, respectively, are indicated by asterisks. Nodes supported by more than 50% of the sampled trees are indicated by a blue bar displaying the 95% credibility intervals of highest posterior density for the node heights. HA-1B clades are defined according to the classification of Anderson et al., 2016 [7]. The boxes on the right grid are colored according to the genogroups defined on genomic segment 4 (HA), 6 (NA) and 7 (M) phylogenies. For segment 6, the boxes were colored in orange for N1 ‘Eurasian avian-like’ lineage, in blue for N2 ‘Scotland-like’ lineage, in yellow for N2 of ‘H3N2 human-like’ lineage, and in green for N2 of ‘Gent-like’ lineage (see Section 3.4.1). For segment 7, the boxes were colored in brown for ‘M ancestral Eurasian avian-like swine’ group, in red for ‘M recent Eurasian avian-like swine’ group, in green for ‘M European swine’ group, in yellow for ‘M Danish-like’ group, and in purple for M segment from the H1N1pdm lineage (see Section 3.4.2).

The first strains belonging to the European H1_hu_N2 lineage that were isolated in France and sequenced dated back to 1997 [24]. Between 1997 and 2006, such H1_hu_Ny strains contained a HA-encoding gene belonging to either clade 1B.1.1 or clade 1B.1.2.3. From 2007, all H1_hu_Ny strains identified in Northwestern France belonged to clade 1B.1.2.3 (Figure 3B and Figure 4). Since 2016, some H1_hu_Ny virus strains were also identified in the north of France but they were found to belong to clade 1B.1.2.1, which groups swIAV strains mostly isolated in other European countries (Figure 3B and Figure 4).

Within the predominant clade 1B.1.2.3, two genogroups named ‘D’ and ‘Δ146-147’, respectively, accumulated nucleotide substitutions and presented specific non-silent mutations (Figure 4). Strains of group D derived from A/Swine/Cotes d’Armor/0113/2006 and circulated in western France between 2006 and 2013 (the 2013 strain was only partially sequenced and was not included in phylogenetic analysis). These strains harbored ten amino acid mutations in their HA protein compared to that of reference strain A/Sw/Scotland/410440/1994 (Scot/94), i.e., H54N, Q68H, K136R, S159R, R163K, T213N, S220T, Y223H, T228I and G254N/S. Four of them, i.e., S159R, S220T, Y223H, and G254N/S, were located in antigenic sites in HA1. In 2006, a reassortant H1_hu_N1 strain from clade 1B.1.2.3 lacked three nucleotides in the HA-encoding gene, leading to the deletion of amino acid K147. In 2011 and 2012, two other H1_hu_N1 strains harbored a HA-1B.1.2.3 gene encoding a HA with two amino acid deletions at positions 146 and 147 (133–134 in H3 numbering), both usually constituting the beginning of the RBS of HA1 (the 2011 strain was only partially sequenced and was not included in phylogenetic analysis). Since then, from 2012 to the end of the study period in 2018, several H1_hu_N2 strains bearing this double deletion and associated mutations were detected in northwestern France, and classified in group ‘Δ146-147’. In addition to the 146-147 amino acid deletions, strains of group ‘Δ146-147’ were characterized by two specific mutations in HA antigenic sites, i.e., V149A and K180M, and others such as A11T, P100S, N104Y, I258M and E291G in HA. Within clade 1B.1.2.3, only two H1_hu_N2 strains harboring the 146-147 deletion in HA did not cluster in the ‘Δ146-147’ group.

The antigenic relationships of 105 H1_hu_Ny viruses sequenced in HA1 were tested in HI assays using a selection of swine antisera produced against virus strains representative of the different HA-1B clades. Whatever the period, antibodies directed against Scot/94 (clade HA-1B.1) retained a good affinity against viruses of clade HA-1B.1.2.3, even stronger than HIS-CA0070/10, HIS-CA0113/06, HIS-22-130212 and HIS-CA0190/06 (Table 3). However, HI titers of HIS-Scot/94 decreased from 2011, which coincided with the emergence of Δ146-147 strains. Thus, this showed a mean HI titer of 113.14 against Δ146-147 strains isolated in 2011–2018, 12-fold weaker than against other contemporary strains from clade HA-1B.1.2.3 excluding those from group D (mean HI titer = 1451.92, Kruskal-Wallis test *p* < 0.001). In contrast, HIS-22-130212 (1B.1.2.3Δ146-147) exhibited three- to four-fold higher HI titers against Δ146-147 strains than HIS-Scot/94. Reactions against strains of group D led to HI titers similar to other strains from clade HA-1B.1.2.3 excluding those of group ‘Δ146-147’ (pairwise Wilcoxon test *p* > 0.1) (Table 3). It could be noted that HI titers of HIS-CA0113/06, although belonging to the 1B.1.2.3-D group, were weaker than those of HIS-Scot/94 against strains of group D isolated after 2011. Finally, HIS containing antibodies directed against H1N1pdm or H1_av_N1 viruses showed no or low HI titers against HA-1B strains, whatever the H1_hu_ clade (Table S3B).

### 3.4. Genetic Evolution of NA-, M- and other Protein-Encoding Genes from H1_av_ (HA-1C) and H1_hu_ (HA-1B) swIAVs

#### 3.4.1. Evolution of NA-Encoding Segments of N1 and N2 Subtypes

Among the 823 H1N1 and 337 H1N2 subtyped strains, complete sequences of NA-encoding genes were obtained for 124 H1N1 strains (115 H1_av_N1 and 9 H1_hu_N1) and 96 H1N2 strains (78 H1_hu_N2 and 18 H1_av_N2).

**Figure 5:**
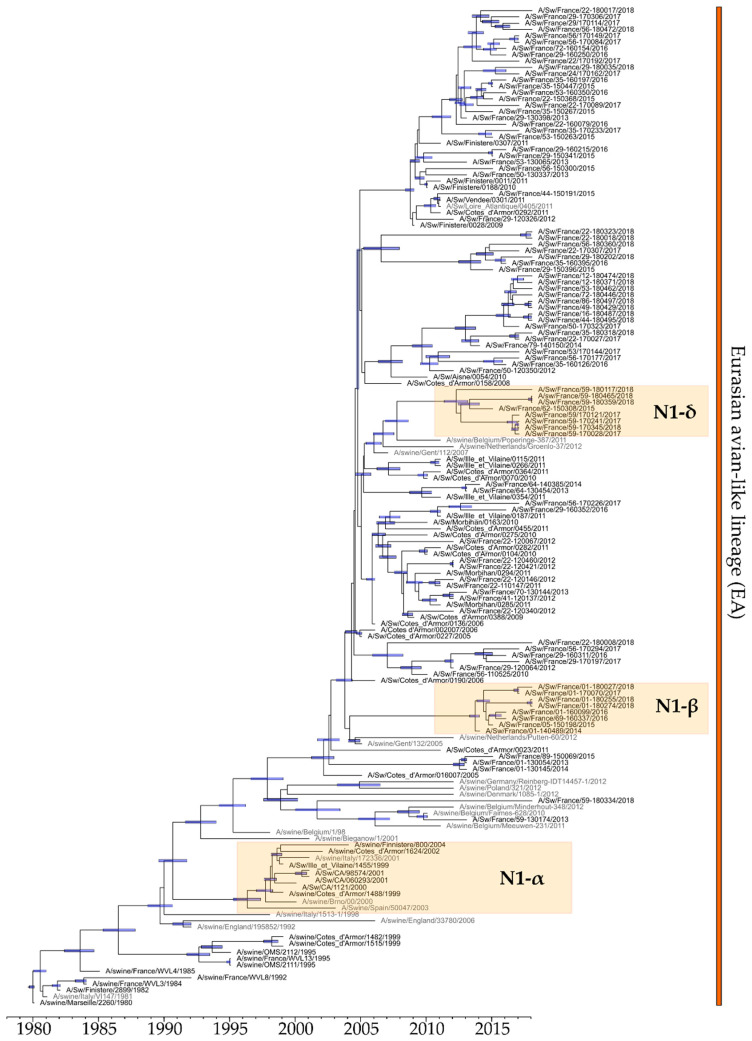
Bayesian inference tree of NA-N1 segment of HxN1 swIAV strains. Strains isolated in pigs in France are indicated in black type, those isolated in pigs from other countries in grey type. Nodes supported by more than 50% of the sampled trees are indicated by a blue bar displaying the 95% credibility intervals of highest posterior density for the node heights. Vertical colored bars in right correspond to the color reported in cases of column #6 in the grids of HA trees (Figure 2 and Figure 4).

All N1 segments of HxN1 swIAVs isolated in France in 2000–2018 derived from the so-called “Eurasian avian-like” (EA) N1 lineage, except those from the H1N1pdm lineage that has been described previously [25] (Figure 5). Most EA-N1 segments were associated with HA-1C genes in H1_av_N1 strains (Figure 2), and only a few with HA-1B gene in H1_hu_N1 reassortants detected from 2005 to 2012 (Figure 4). Congruently with H1_av_ phylogeny, the strains from HA-1C.2.3, 1C.2.1-B and 1C.2.1-C phylogenetic groups formed three clusters within the EA-N1 lineage, mentioned as N1-α, -β and -δ, respectively (Figure 5). Comparison of deduced amino acid sequences revealed that NA genes in group N1-α were distinguished from other N1-encoding sequences by 25 group-specific mutations of which four were located in sites predicted to be antigenic determinants for NA, i.e., S339F, H341N, V394I and I396M. In N1-β group, the encoded NA were characterized by 16 amino acid mutations including three in sites described as antigenic determinant, i.e., R331G, H341N/D and T397N/S, and one mutation creating an additional glycosylation site, NA-S88N. Strains belonging to the N1-δ group were characterized by seven mutations including N/S385K in a predicted antigenic site. No compensatory mutation was identified in N1 amino acid sequences to optimize HA/NA balance when they were associated with a HA-1B in reassortant H1_hu_N1 strains.

**Figure 6:**
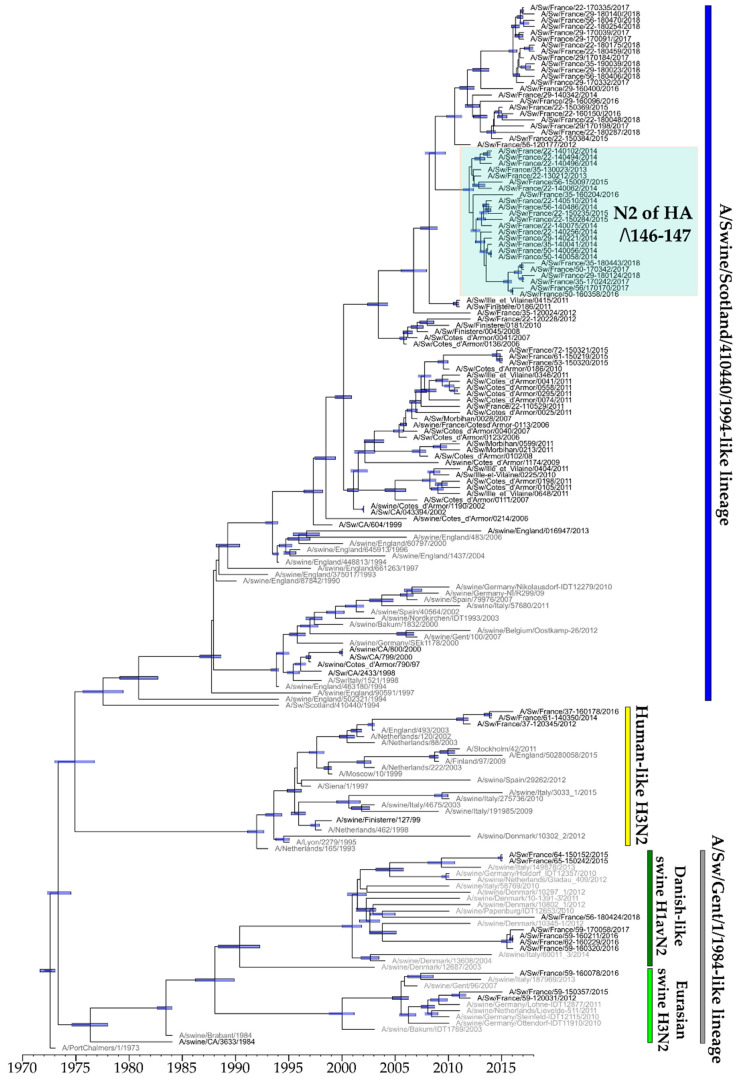
Bayesian inference tree of NA-N2 segment of HxN2 swIAV strains. Strains isolated in pigs in France are indicated in black type, those isolated in humans or pigs in other countries in grey type. Nodes supported by more than 50% of the sampled trees are indicated by blue bar displaying the 95% credibility intervals of highest posterior density for the node heights. Vertical colored bars at the right correspond to colors reported in cases of column #6 in the grids of HA trees (Figure 2 and Figure 4).

The N2 segments sequenced in this study were associated either with H1_hu_ (HA-1B) or H1_av_ (HA-1C) genes in H1_hu_N2 or H1_av_N2 strains, respectively (Figure 2 and Figure 4). Phylogeny of N2 segment exhibited more genetic diversity than those for N1 segments as they classified into three distinct lineages (Figure 6). The majority of H1N2 strains grouped in the “A/Sw/Scotland/410440/1994-like lineage” which derived from the original H1_hu_N2 strain in Europe (Figure 6). Within this N2 “Scotland-like” lineage, strains harboring H1_hu_ Δ146-147 genes formed a cluster and were characterized by two specific amino acid mutations in N2, i.e., I50M and V317I. A second N2 lineage derived from human-like reassortant swine H3N2 strains belonging to the “A/Gent/1/1984-like lineage”. This N2 “Gent-lineage” comprised H1_hu_N2 strains isolated since 2016 in the northern part of France, as well as reassortant H1_av_N2 strains with a HA-1C.2. Within this lineage, French H1N2 strains had N2 genes closer to those described in Danish enzootic H1_av_N2 viruses than to those of Eurasian swine H3N2 viruses (Figure 6). Finally, three reassortant H1_av_N2 strains shared a N2 gene derived from a human seasonal H3N2 lineage, as detailed in a previous study [44] (Figure 6).

Analysis of residues involved in IAV resistance to antiviral drugs identified only two H1_av_N1 strains that each naturally acquired a mutation, assessed to impact the efficacy of NA inhibitors, NA-Y155H classified as highly reduced inhibition (HRI) to oseltamivir and zanamivir, and NA-S247N classified as reduced inhibition (RI) to oseltamivir for H5N1 viruses, according to the list of the WHO [45]. By contrast, 80% of H1_hu_N2 strains harbored mutation NA-S331R, also classified as RI to oseltamivir and zanamivir [45]. The H1_av_N2 strains with N2 of human seasonal origin were the only ones harboring mutation NA-N329K classified RI to oseltamivir and zanamivir [45]. Finally, mutations Y347K and V149I in HxN1 strains and mutation K249R in HxN2 strains were located in amino acid sites described as impacting the efficiency of NA inhibitors, but their resistance phenotype has never been tested.

#### 3.4.2. Evolution of M-Encoding Segment

**Figure 7:**
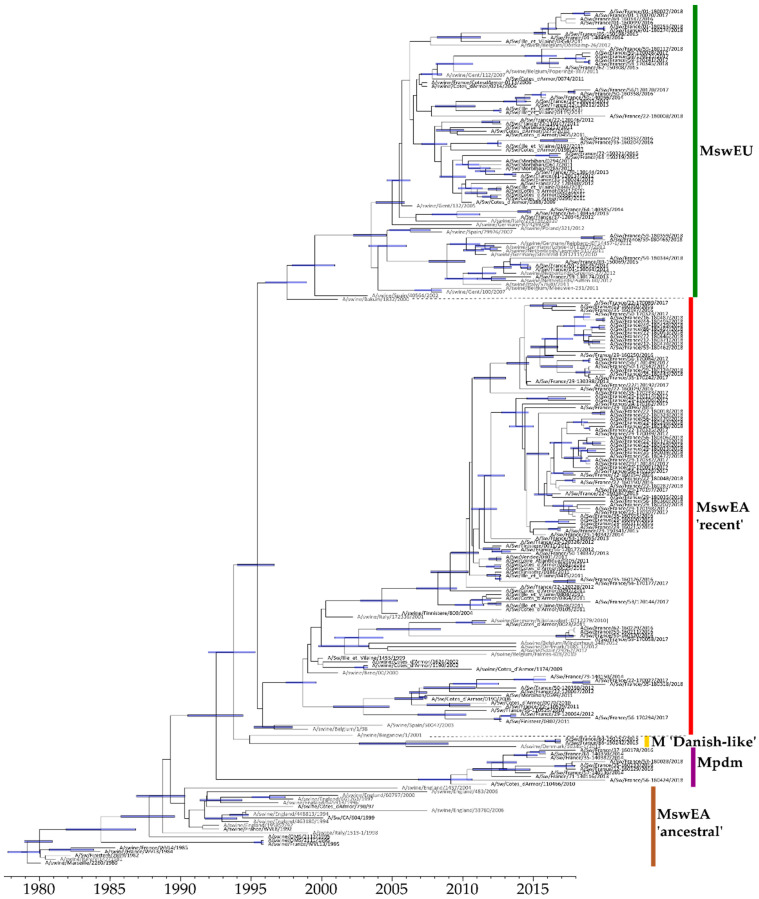
Bayesian inference tree of M-gene segment of H1Ny viruses. Strains isolated in pigs in France are indicated in black type, those isolated in pigs from other countries in grey type. Nodes supported by more than 50% of the sampled trees are indicated by blue bar displaying the 95% credibility intervals of highest posterior density for the node heights. Vertical colored bars at the right correspond to M genogroups with the colors reported in cases of column #7 in the grids of HA trees (Figure 2 and Figure 4).

Complete sequences of M-encoding genes were obtained for 170 H1Ny strains (100 H1_av_N1, 12 H1_av_N2, 54 H1_hu_N2 and 4 H1_hu_N1) isolated from 2000 to 2018 and included in the analysis. The M-encoding genes of French swine H1Ny strains segregated into several genogroups (Figure 7), that were defined from a large scale phylogenetic analysis (Figure S1). Two H1_av_N2 strains with a HA-1C.2 harbored a M segment closer to those of Danish enzootic H1_av_N2 strains (M ‘Danish-like’ group) than of other H1Ny strains isolated in France, and congruent with the NA segment. Three reassortant H1_av_N2 strains harbored a M segment from the H1N1pdm lineage (Mpdm). Two of these, with a N2 from human seasonal IAV, acquired only the M segment from the H1N1pdm, whereas the third one harbored all internal segments from the H1N1pdm lineage, as detailed in previous studies [44,46]. Finally, the majority of French swine H1Ny strains classified into two main genogroups we called the “M recent Eurasian avian-like swine” group (MswEA-‘recent’) and the “M European swine” group (MswEU), respectively, which showed no specific associations with HA or NA clades (Figure 2 and Figure 4).

All these M genogroups were distinguished mainly by specific mutation patterns in matrix protein 2 (M2), especially in its extracellular domain M2e and the transmembrane helices (TM) (data not shown). MswEU and MswEA-‘recent’ shared the known adamantane resistance mutation M2-S31N, but MswEU differed by mutations M2-R18K and -D21G in M2e and four mutations in TM, including two in adamantane resistance sites, i.e., -L26I and -V27L [47,48].

#### 3.4.3. Evolution of Other Internal Protein-Encoding Genomic Segments

Phylogenetic analyses of polymerase (PB2, PB1 and PA), nucleoprotein (NP) and non-structural (NS) protein-encoding segments of H1_av_ and H1_hu_ swIAVs identified in France until the end of 2018 showed that they all derived from the “Eurasian avian-like swine” lineage whatever the H1 lineage or clade (data not shown), excluding the reassortant strain with internal segments from H1N1pdm.

The deduced amino acid sequences of the internal proteins of the H1_av_ and H1_hu_ swIAVs were overall conserved, with a mean entropy value of 0.077, considering that a residue entropy exceeding 1.0 is highly variable and the maximum value is 4.322 (log_2_ 20). In any case, the PB1-F2, PA-X, M2 and NS1 proteins showed the greatest residue variability with less than 50% of conserved residues and mean entropy values that were 1.5- to 5-fold higher than the overall mean (Table 4).

Added to their weak residue conservation (8.9%) (Table 4), the PB1-F2 proteins presented a wide variation in length. Almost 40% of the 162 amino acid sequences deduced for strains isolated from 2000 to 2018 were truncated before the 90th amino acid, and harbored a stop codon at positions 9 (0.6%), 12 (3%), 26 (5.6%), 58 (18.5%), 80 (5.6%) or 88 (6.2%) after the methionine. PB1-F2 proteins of strains with an H1_av_ belonging to HA-1C.2.3 clade were all truncated at position 12, those with HA-1C.2.2 and HA-1B.1.2.1 at position 80, and those with HA-1C.2.1-B at position 88. Three H1_av_N1 strains from HA-1C.2.1 clade (1.8%) probably did not even encode PB1-F2 protein since they had a threonine instead of a methionine at the first position.

Almost all French swIAVs presented the PA-X full length pattern that was associated with highly virulent strains [49]. Only three strains (1.8%) of H1_hu_N1, H1_hu_N2 and H1_av_N2 lineages, respectively, had a PA truncated at residue 42 in the X-domain, as in PA from H1N1pdm strains. Moreover, seven strains were found to have additional amino acids as compared to those encoded by the commonly described full-length 61-codon X-ORF. Five of them, including the three reassortant H1_av_N2 strains with a N2 from seasonal human IAV, harbored a base substitution that transformed the stop codon into a cysteine codon at position 253 followed by seven additional residues (MPGLNHS). Two other strains, of H1_hu_N2-Δ146-147 and H1_av_N1 lineages, had a leucine codon at position 253 and encoded three or seven additional residues (MPG or MGPLNHS), respectively.

Comparison of amino acid sequences, as well as research of molecular markers for virulence, allowed the identification of mutations in internal segments that were statistically linked to HA gene phylogenetic clusters. In addition to PB1-F2 truncation, all the H1_av_ strains from HA-1C.2.1-B clade and the two H1_av_N2 strains from HA-1C.2 clade isolated in 2015 harbored the mutation N66S in PB1-F2, described as a virulence marker in several IAV subtypes [50]. H1_av_ strains from HA-1C.2.1-B clade were also characterized by 40 other co-occurring amino acid changes in internal proteins (Table S4), including mutation D92E in NS1 that was suspected of conferring virulence and resistance to IFN-α of H5N1 virus strains in pigs [51]. H1_av_ strains from HA-1C.2.1-A clade were characterized by ten co-occurring mutations (Table S4), including PB1-F2-E87G described to decrease lethality of H5N1 strains in ducks [50]. H1_av_ strains from HA-1C.2.1-C clade were characterized by 29 co-occurring mutations (Table S4), including PB1-S654N and PA-T343A that were associated with a poor fitness of A/Puerto Rico/8/34(H1N1) and H1N1pdm virus strains, respectively [52,53]. H1_av_ strains from 1C.2.2 clade were characterized by PB1-K353R and PA-S409N mutations reported to increase virulence and transmission of several IAV subtypes [54,55]. Finally, within the H1_hu_ lineage, strains from clade 1B.1.2.1, isolated in the northern France, were characterized by two mutations in NP that were described as increasing virulence and resistance to MxA. The first, mutation NP-Y289H, was also observed in reassortant H1_av_N2 strains with a N2 from seasonal human IAV. The second mutation, NP-Q357K, was also observed in H1_av_ strains isolated in the same geographic area [56,57].

### 3.5. Overview of H1_av_ and H1_hu_ Genotypes Identified in France from 2000 to 2018

Taking into account the clade and/or lineage identified for each genomic segment, 17 distinct genotypes were identified from the 161 H1_av_ and H1_hu_ swIAV strains fully sequenced in this study (Table 5). Among them, 16 genotypes covered eight genotypes previously described by Watson et al. ([13], Table 5).

## 4. Discussion

SwIAV strains of European enzootic H1_av_N1 and H1_hu_N2 lineages were quickly introduced into the pig population in France after their emergence in 1979 and 1994, respectively [8,9,10]. Between 2000 and 2010, the epidemiological surveillance of swIAVs was poorly organized and few samples were collected. The IAV pandemic in 2009 led to the implementation of a national surveillance network which, combined with improvement of molecular subtyping and sequencing techniques, has permitted the production of a sharper picture of the evolution, diversity and distribution of swIAV strains in France.

H1_av_N1 was predominant in France between 2000 and 2018 (66.5%), as in most European countries except the United Kingdom and Denmark, according to the European studies conducted between 2010 and 2017 [12,13,21]. The H1_av_Ny strains identified in pigs in France clustered mainly in the HA clade 1C.2.1, described in Europe and Russia [7]. Only a few strains isolated in the East of the country carried an HA from clade 1C.2.2, which was detected in other European countries such as Germany, Italy, Luxembourg, Netherlands, Poland and Spain [7]. Three H1_av_N2 strains with an HA belonging to the 1C.2 clade, similar to contemporary strains circulating in Denmark, were detected in France. The first two were observed in Southwestern France in spring 2015 and were close to the “Danish-like lineage” in all other segments, whereas the third was detected in Northwestern France in 2018 and had internal segments of H1N1pdm [46,58]. These sporadic detections during the study period sustain the hypothesis that these strains were introduced thanks to live pig importations from abroad.

H1_hu_N2 was the second most frequent subtype (25.4%) detected in pigs in France in 2000–2018. Its proportion was similar to those observed between 2010 and 2013 in Italy, Spain and Netherlands, but twice than that reported in the United Kingdom and Germany at that time [12,18]. However, European H1_hu_Ny subtypes classified in different HA clades according to their geographical location [7]. In France, between 1997 and 2016, viruses from the H1_hu_N2 subtype were only detected in the north-west. They belonged mainly to the HA-1B.1.2.3 clade, which was found only in France, in association with a NA segment from the A/Sw/Scotland/410440/1994-like lineage. From 2017, new H1_hu_N2 strains were detected in the north of France. They harbored an H1 from 1B.1.2.1 clade and a NA segment from A/Sw/Gent/1984-like lineage, similarly to strains described in Belgium, Germany, Netherlands and Denmark [7,13]. Commercial exchanges and human movements between these border countries could explain the presence of this lineage in this part of France.

Within the HA-1B.1.2.3 clade, several groups of strains diverged from A/Sw/Cotes d’Armor/790/97 but co-circulated in the same geographical area. The more recent strain belonging to group D was detected in 2013, which suggests that this group is rare or no longer present in the territory, maybe due to a lack of competitiveness with other strains from HA-1B.1.2.3 clade. Interestingly, most strains since 2012 harboring the double 146-147 deletion and associated mutations in HA also formed a phylogenetic cluster. A specific RT-qPCR was developed by the French NRL for to discriminate H1_hu_ Δ146-147 within H1_hu_ strains [26]. This tool revealed that H1_hu_ Δ146-147 strains quickly spread until reaching approximately 54% of H1_hu_ viruses detected in pigs in France in 2013–2014, a proportion that further decreased to 18% in 2018 (data not shown). Interestingly, the same deletion into the RBS has repeatedly been described in swIAV isolates and observed within various HA clades: HA-1B.1.2.2 in Italy [59], HA-1B-Other-human clade in Russia [60] and the “classical swine clade” HA-1A.1.3 (H1α3) in Canada [61]. One of the two residues, either 146 or 147, was also deleted in the three HA-1C.2 strains identified in France. Fixation of the same amino acid deletions into RBS of HA from the three 1A, 1B and 1C lineages is not yet explained but has been investigated elsewhere to understand the impact on virus fitness, pathogenicity and immune escape [62].

Interestingly, the regionalization of HA clades, either of H1_av_ or H1_hu_ lineages, could be explained by the limited number of pig movements through the French territory as compared to other countries. The majority of farms perform farrow-to-finish production, and the leading destination of movements is slaughterhouses/rendering plants (75.2%), followed by exchanges between farms (22.8%) and trade operators (2.0%) [63]. Movements of live pigs between European countries in both import and export represent only 1.4% of transport rounds [63].

Antigenic drift for swIAVs was previously estimated to be of 0.15 and 0.17 antigenic units (AUs) per year for H1_av_ and H1_hu_ lineages, respectively [64], which is weak compared to human IAV strains (0.6 to 1.2 AU/year [65,66]). Assuming that an antigenic distance above 2 AU, or a four-fold difference in HI assay titers, is significant and considered as an antigenic drift [67], it was expected that genetic swIAV evolution would generate a significant antigenic drift every 12–13 years. In the present study, such an antigenic distance was observed between the newly introduced H1_av_ strains HA-1C.2 and the predominant strains HA-1C.2.1, as well as between the H1_hu_ Δ146-147 strains and other H1_hu_ strains. However, a slight stepwise antigenic drift could be noted within H1_av_ 1C.2.1 lineage every four years, which could be associated with specific mutations in RBS and antigenic sites, e.g., HA-N142D and K180N, rather than gradual accumulation of mutations. Such punctual mutations in HA as those described in this study could lead swIAV to escape vaccine protection quicker than expected, suggesting that vaccine antigens should be regularly and/or locally updated. In France, vaccination is applied in a little less than half of the breeding herds [22]. During the study period, the adjuvanted inactivated trivalent vaccine Respiporc FLU3^®^ (Ceva, Libourne, France, formerly IDT-Biologika, Dessau-Roßlau, Germany) was the only vaccine that has been issued [22]. Respiporc FLU3^®^ contains antigens representative of European H1_av_N1, H1_hu_N2, and H3N2 lineages, which have not been changed since the vaccine has been launched in 2010.

Exchange of HA- and NA-encoding segments were regularly observed during the studied period. However, H1_hu_N1 viruses (*n* = 7) have seemed rarer than H1_av_N2 viruses (*n* = 25) since 2010. The co-evolution of HA and NA segments, linked to the maintenance of the HA-NA balance [68], may explain in part the limited number of viruses that exchanged their glycoproteins. Among H1_av_N2, N2 segment was generally provided by enzootic H1_hu_N2 viruses (N2-Scotland lineage) but not swine H3N2 viruses (N2-Gent lineage), in line with their frequency and area of co-circulation with H1_av_N1 viruses. Indeed, swine H3N2 subtype is rare in France, with less than one isolate detected each year on average, and furthermore restricted to the north (data not shown), which limits reassortment opportunities. However, three H1_av_N2 strains were shown to contain a N2 gene from a seasonal human H3N2 lineage, recalling the importance of implementing biosecurity measures in holdings to avoid inter-species transmissions that may lead to the emergence of new viruses in pigs [44].

In contrast to other European countries in which swIAV diversity has been strongly impacted by the transmission of the H1N1 pandemic virus to pigs in 2009, the genotype diversity of French swIAVs has not changed greatly due to this new introduction between 2009 and 2018 [13,15,16,17,18,19]. The H1_av_ and H1_hu_ viruses were widely established for years in northwestern France and might have hampered the H1N1pdm spread in French herds located in this high pig density area [22,25]. Thus, co-infections of pigs with H1_av_ or H1_hu_ strains and H1N1pdm virus would have remained a rare event, which would explain the limited number of reassortant strains between these lineages. Only three H1_av_N2 strains harboring internal segments from the H1N1pdm lineage have been identified in our study. Given their detailed genotypes, two of them, containing only the Mpdm, would have been generated on the territory [44]. However, the third, with a HA from clade 1C.2, a N2-Gent and all the internal genes from H1N1pdm, would have been introduced in Brittany in toto from abroad in 2018 [46]. Four other reassortant strains of H1_av_N1 subtype, containing one or several internal segments from H1N1pdm, have been recently reported using SYBR-Green RT-qPCRs [21]. Thus, at least three additional genotypes would complete the list of the seventeen we have discriminated here. Whereas these reassortant detections have remained sporadic, the risk of reassortment events could increase in the near future, as the number of H1N1pdm virus strains detected in France has increased since 2016 [25,44].

Except for three reassortant strains, internal segments of H1Ny viruses belonged to the Eurasian avian-like (EA) lineage and showed a weak genetic evolution. However, we highlighted the co-occurrence of two M clades within the EA lineage in France and elsewhere in Europe. Before the emergence of the H1N1pdm virus, Furuse and coll. identified only one M gene swine lineage in Europe [36]. This lineage was maintained in European and Asian swIAVs, but it appears that a new lineage derived in Europe and spread in all swIAV subtypes from 2002. The two M clades, swEA and swEU, were identified in French swIAVs regardless of region or lineage. The M swEU clade presented specific mutations in the ectodomain (M2e) and transmembrane domain of the matrix protein M2. However, the origin and the consequences of the emergence of this new M clade are poorly understood up to now. The M2e domain has heretofore been considered to be host-specific and invariable between IAV subtypes, suggesting that antibodies against M2e might confer cross-protection which is currently being explored for a universal IAV vaccine development [69,70,71]. However, the variability in the M2e domain, such as here observed in swIAVs could help virus strains to evade the immune system, raising questions about the accuracy of such M2e-based vaccines.

Finally, the analysis of the internal protein-encoding genes showed a variability in length for PB1-F2 and PA-X, and detected several mutations suspected of altering the virulence of strains. Thus, the H1_av_N1 strains of clade HA-1C.2.1 group B harbored a truncated PB1-F2 protein and mutations PB1-F2-N66S and NS1-D92E were described as increasing virulence [50,72,73,74]. However, these strains remained located in the Eastern part of France, in an area with a low pig density, which may have limited their spread.

## 5. Conclusions

To conclude, the genetic evolution of swIAVs in France in the last two decades is characterized by geographical divergence in H1_av_Ny viruses and apparition of H1_hu_ Δ146-147 strains, which entailed the adaptation of RT-PCR primers for HA subtyping. Besides marked antigenic drift in H1_hu_ Δ146-147 strains, some amino acid mutations in HA induced slight antigenic drifts every four years within H1_av_ and H1_hu_ lineages, which would require the reference antigens to be used in HI tests for serological investigations to be re-evaluated regularly. The low number of samples taken in some areas of France (the center and the East) do not preclude the presence of additional minor H1 genotypes in the French territory. Thus the diversity could be even greater than observed. The genotype constellation was widely dominated by H1_av_N1 and H1_hu_N2 strains, with internal segments of the Eurasian avian-like lineage, between 2000 and 2018. However, the situation might evolve with further adaptation of new variant or reassortant viruses, in line with increasing frequency of H1N1pdm infections and/or new virus spreading following imports. Finally, the identification of molecular markers for virulence and antiviral resistance also recalls the importance of implementing strategies to disrupt swIAV spreading within and between pig herds, as well as IAV transmissions between humans and pigs.

## Figures and Tables

**Table 1:** Influenza A virus (IAV) strains inoculated to specific pathogen-free (SPF) pigs for production of swine antisera.

Strain (Abbreviated Name)	Subtype	Swine H1 Clade [7]
A/Swine/Finistere/2899/82 (Fin2899/82)	H1_av_N1	1C.1
A/Swine/Morbihan/0070/05 (Mo0070/05)	H1_av_N1	1C.2.1
A/Swine/Cotes d’Armor/0388/09 (CA0388/09)	H1_av_N1	1C.2.1
A/Swine/Cotes d’Armor/0186/10 (CA0186/10)	H1_av_N2	1C.2.1
A/Swine/France/65-150242/15 (65-150242)	H1_av_N2	1C.2
A/Swine/Scotland/410440/94 (Scot/94)	H1_hu_N2	1B.1
A/Swine/Cotes d’Armor/0214/06 (CA0214/06)	H1_hu_N2	1B.1.1
A/Swine/Cotes d’Armor/0113/06 (CA0113/06)	H1_hu_N2	1B.1.2.3
A/Swine/France/22-130212/13 (22-130212)	H1_hu_N2	1B.1.2.3 (∆146-147) *
A/Swine/Cotes d’Armor/0070/10 (CA0070/10)	H1_hu_N1	1B.1.2.3
A/Swine/Cotes d’Armor/0190/06 (CA0190/06)	H1_hu_N1	1B.1.2.3 (∆147) *
A/Swine/England/117316/86	clswH1N1	1A.1-like
A/California/04/09	H1N1pdm	1A.3.3.2
A/Swine/Sarthe/0255/10	H1N1pdm	1A.3.3.2

* ∆146-147 and ∆147 refer to amino acid deletions in hemagglutinin (HA) at positions 146 and/or 147 after the first methionine including the signal peptide.

**Table 2:** Cross-hemagglutination inhibition (HI) data obtained for H1_av_Ny swIAVs isolated in France in 2000–2018 against hyperimmune swine antisera directed towards HA-1C representative strains. The table reports the HI titers for reference antigens (homologous HI titers are underlined), then the geometric mean HI titers +/− the standard deviation (in bold) obtained by strains grouping in the different HA-1C (H1_av_) clades over time. Results of the Wilcoxon pairwise comparison test are reported by a letter in superscript, a different letter indicating a significant difference between groups in the row and column (*p* < 0.05). HI titers of subgroups within 1C.2.1 were tested independently of the other rows. HI titers for HA-1C.2 and 1C.2.2 clades were not included in the statistical test because the sample sizes were below five. The range of HI antibody titers obtained within each group of tested strains is reported in square brackets with the number of tested strains in italics. Raw data for individual studied strains are available upon request.

		Hemagglutination Inhibition Titer to Pig Antiserum Produced against Selected H1_av_ Isolates				
HA Clade	swIAV Strains (Name or Period of Isolation or Subgroup Within 1C.2.1 Clade of Tested Strains)	A/Sw/Finistere/2899/82 (1C.1-H1_av_N1)	A/Sw/Morbihan/0070/05 (1C.2.1-H1_av_N1)	A/Sw/Cotes d’Armor/0388/09 (1C.2.1-H1_av_N1)	A/Sw/Cotes d’Armor/0186/10 (1C.2.1-H1_av_N2)	A/Sw/France/65-150242/15 (1C.2-H1_av_N2)
1C.1	A/Sw/Finistere/2899/82	2560	80			
1C.2.1	A/Sw/Morbihan/0070/05	40	640			
	A/Sw/Cotes d’Armor/0388/09		320	640		
	A/Sw/Cotes d’Armor/0186/10		320	320	320	
1C.2	A/Sw/France/65-150242/15			80	<10	2560
1C.2.1	2000–2010	**215.34 ± 2.85 ^a^**	**490.23 ± 1.62 ^a^**	**525.01 ± 1.69 ^a^**		
		[40–640]	[160–1280]	[320–1280]		
		*16*	*26*	*7*		
	2011–2014		**750.18 ± 1.58 ^b^**	**879.33 ± 1.83 ^b^**	**242.51 ± 1.81 ^c^**	
			[320–1280]	[320–2560]	[<10–640]	
			*48*	*48*	*30*	
	2015–2018			**336.99 ± 1.96 ^d^**	**132.81 ± 1.71 ^e^**	**16.59 ± 3.17 ^f^**
				[40–1280]	[80–320]	[<10–80]
				*58*	*58*	*36*
1C.2	2015–2018			**40 ± 3.32**		**320 ± 18.93**
				[10–80]	[<10]	[40–2560]
				*3*	*3*	*2*
1C.2.2	2013–2015			**160 ± 2.67**	**113.14 ± 1.63**	
				[80–320]	[80–160]	
				*2*	*2*	
Subgroups within 1C.2.1 2015–2018	group A			**427.15 ± 1.84 ^g^**	**146.72 ± 1.8 ^ij^**	**30.07 ± 1.74 ^k^**
				[160–1280]	[80–320]	[10–80]
				*22*	*22*	*15*
	group B			**246.75 ± 1.43 ^h^**	**113.14 ± 1.45 ^i^**	**9.56 ± 3.66 ^m^**
				[160–320]	[80–160]	[<10–20]
				*6*	*6*	*4*
	group C			**470.32 ± 1.44 ^g^**	**201.59 ± 1.63 ^j^**	**26.92 ± 1.45 ^k^**
				[320–640]	[80–320]	[20–40]
				*8*	*8*	*6*
	Others (not A, B or C)			**265.52 ± 2.17 ^h^**	**110.16 ± 1.57 ^i^**	**7.7 ± 4.11 ^m^**
				[40–1280]	[<10–320]	[<10–40]
				*22*	*22*	*11*

**Table 3:** Cross-hemagglutination inhibition (HI) data obtained for H1_hu_Ny swIAVs isolated in France in 2000–2018 against hyperimmune swine antisera directed towards HA-1B representative strains. The table reports the HI titers for reference antigens (homologous HI titers are underlined), then the geometric mean HI titers +/− the standard deviation (in bold) obtained by strains grouping in the different HA-1B (H1_hu_) clades over time. The second part of the table reports the detailed titers for subgroups within the HA-1B.1.2.3 clade over time. Results of the Wilcoxon pairwise comparison test are reported by a letter in superscript; a different letter indicates a significant difference between groups in row and column (*p* < 0.05). HI titers of subgroups within 1B.1.2.3 were tested independently of the first part of the table. The groups with sample sizes below five were not included in the statistical test. The range of HI antibody titers obtained within each group of tested strains is reported in square brackets with the number of tested strains in italics. Raw data for individual studied strains are available upon request.

HA Clade	swIAV Strains (Name or Period of Isolation or Subgroup within 1B.1.2.3 Clade of Tested Strains)	Hemagglutination Inhibition Titer to Pig Antiserum Produced Against Selected H1_hu_ Isolates					
		A/Sw/Scotland/ 410440/94 (1B.1-H1_hu_N2)	A/Sw/Cotes d’Armor/0214/06 (1B.1.1-H1_hu_N2)	A/Sw/Cotes d’Armor/0070/10 (1B.1.2.3-H1_hu_N1)	A/Sw/Cotes d’Armor/0113/06 (1B.1.2.3-D H1_hu_N2)	A/Sw/France/ 22-130212/13 (1B.1.2.3Δ146-147 H1_hu_N2)	A/Sw/Cotes d’Armor/0190/06 (1B.1.2.3Δ147 H1_hu_N1)
1B.1	A/Sw/Scotland/410440/94	2560	320		160		10
1B.1.1	A/Sw/Cotes d’Armor/0214/06	320	1280		160		10
1B.1.2.3	A/Sw/Cotes d’Armor/0070/10	1280		640			
	A/Sw/Cotes d’Armor/0113/06	1280	320		1280		20
	A/Sw/France/ 22-130212/13	160	320	320	40	1280	160
	A/Sw/Cotes d’Armor/0190/06	40	10	20	<10		1280
1B.1.1	2000–2010	**320 ± 2.4 ^a^** [80–1280] *6*	**640 ± 1.63 ^a^** [320–1280] *5*		**100.79 ± 1.76 ^f^** [40–160] *6*		**4.57 ± 4.12 ^e^** [<10–20] *5*
1B.1.2.3	2000–2010	**1539.87 ± 1.63 ^b^** [640–2560] *15*	**359.19 ± 1.92 ^a,d^** [80–640] *12*	**452.55 ± 1.63** [320–640] *2*	**525.01 ± 1.88 ^d^** [160–1280] *14*		**40 ± 2.06 ^b^** [10–80] *12*
	2011–2014	**510.37 ± 3.87 ^a^** [40–5120] *49*	**187.44 ± 3.44 ^d^** [<10–1280] *38*	**240.8 ± 3.38 ^d^** [20–1280] *39*	**61.53 ± 6.27 ^f^** [<10–640] *50*	**320 ± 2 ^a,c^** [160–1280] *11*	**69.59 ± 3.88 ^f^** [<10–1280] *38*
	2015–2018	**499.65 ± 4.22 ^a,d^** [20–2560] *28*	**160 ± 1.76** [80–320] *4*	**342.97 ± 3.27 ^d^** [20–1280] *30*	**77.02 ± 4.81 ^f^** [<10–320] *30*	**201.59 ± 2.23 ^c^** [40–640] *30*	**134.54 ± 2.39** [40–320] *4*
1B.1.2.1	2015–2018	**2031.87 ± 1.49** [1280–2560] *3*		**806.35 ± 1.49** [640–1280] *3*	**253.98 ± 1.49** [160–320] *3*	**126.99 ± 1.49** [80–160] *3*	
Subgroups within 1B.1.2.3 2000–2010	All except groups D	**1741.81 ± 1.65 ^g^** [640–2560] *9*	**390.08 ± 2.16 ^i^** [80–640] *7*		**452.55 ± 1.9 ^i^** [160–1280] *8*		**65.63 ± 1.4 ^p^** [40–80] *7*
	group D	**1280 ± 1.55 ^g^** [640–2560] *6*	**320 ± 1.63 ^i^** [160–640] *5*		**640 ± 1.86 ^g,i^** [320–1280] *6*		**20 ± 1.63 ^n^** [10–40] *5*
Subgroups within 1B.1.2.3 2011–2014	All except groups D & Δ146-147	**1612.7 ± 1.66 ^g^** [640–5120] *21*	**452.55 ± 1.66 ^i^** [160–1280] *16*	**728.82 ± 1.68 ^j^** [320–1280] *16*	**181.49 ± 1.94 ^m^** [40–640] *22*	**190.27 ± 1.41** [160–320] *4*	**39.45 ± 3.07 ^p^** [<10-160] *16*
	group D	**829.98 ± 2.84 ^g^** [80–2560] *8*	**12.65 ± 36.19** [<10–160] *2*	**160 ± 6.26** [20–640] *3*	**226.27 ± 2.67 ^i,m^** [40–640] *8*		**3.16 ± 5.09** [<10–10] *2*
	Δ146-147	**125.53 ± 1.76 ^h^** [40–320] *20*	**121.26 ± 1.99 ^h^** [40–320] *20*	**105.56 ± 2.21 ^h^** [20–320] *20*	**11.12 ± 5.17 ^k^** [<10–40] *20*	**430.69 ± 1.97 ^j^** [160–1280] *7*	**149.29 ± 2.1 ^h^** [40–1280] *20*
Subgroups within 1B.1.2.32015–2018	All except group Δ146-147	**1280 ± 1.79 ^g^** [320–2560] *18*		**685.94 ± 1.81 ^j^** [160–1280] *20*	**190.27 ± 1.72 ^m^** [80–320] *20*	**144.2 ± 1.98 ^m^** [40–320] *20*	
	Δ146-147	**91.9 ± 2.2 ^h^** [20–320] *10*	**126.99 ± 1.49** [80–160] *3*	**85.74 ± 2.14 ^h^** [20–160] *10*	**12.62 ± 3.92 ^k^** [<10–40] *10*	**393.97 ± 1.77 ^j^** [160–640] *10*	**201.59 ± 1.49** [160–320] *3*

**Table 4:** Residue variability for deduced amino acid sequences of internal proteins for H1_av_ and H1_hu_ swIAVs isolated in France in 2000–2018.

SwIAV Proteins	PB2	PB1	PB1-F2	PA	PA-X	NP	M1	M2	NS1	NS2
Protein length (in amino acid)	759	757	90	716	260	498	252	97	230	121
Nb of conserved residues (%)	590 (77.7%)	552 (72.9%)	8 (8.9%)	521 (72.8%)	158 (60.8%)	406 (81.5%)	211 (83.7%)	48 (49.5%)	98 (42.6%)	82 (67.8%)
Mean Entropy	0.055	0.068	0.379	0.062	0.117	0.040	0.019	0.157	0.187	0.082
Max. Entropy	1.273	1.318	1.827	1.391	1.268	0.986	0.711	1.493	1.419	1.016
Entropy st. dev.	0.159	0.176	0.364	0.168	0.236	0.123	0.065	0.307	0.274	0.178

**Table 5:** Genotypes identified for H1_av_ (HA-1C) and H1_hu_ (HA-1B) swIAVs isolated in France from 2000 to 2018.

Subtype	Protein-Encoding Genomic Segment								Frequency Nb (%)	Corresponding Genotype Previously Described in [13] *
	HA [7]	NA	PB2	PB1	PA	NP	M	NS		
**H1_av_N1**	**1C.2.1**	N1-EA	EA	EA	EA	EA	swEA	EA	34 (21.1)	A
	1C.2.1	N1-EA	EA	EA	EA	EA	swEU	EA	55 (34.2)	A
	1C.2.2	N1-EA	EA	EA	EA	EA	swEA	EA	3 (1.9)	A
	1C.2.3	N1-EA	EA	EA	EA	EA	swEU	EA	2 (1.2)	A
H1_av_N2	1C.2	N2-Gent	EA	EA	EA	EA	Dk-like	EA	2 (1.2)	D
	1C.2	N2-Gent	pdm	pdm	pdm	pdm	pdm	pdm	1 (0.6)	T
	1C.2.1	N2-Scot	EA	EA	EA	EA	swEA	EA	3 (1.9)	G
	1C.2.1	N2-Scot	EA	EA	EA	EA	swEU	EA	2 (1.2)	G
	1C.2.1	N2hu	EA	EA	EA	EA	swEA	EA	1 (0.6)	I
	1C.2.1	N2hu	EA	EA	EA	EA	pdm	EA	2 (1.2)	Not described
H1_hu_N2	1B.1.1	N2-Scot	EA	EA	EA	EA	swEA	EA	1 (0.6)	C
	1B.1.2.3	N2-Scot	EA	EA	EA	EA	swEU	EA	29 (18.0)	C
	1B.1.2.3	N2-Scot	EA	EA	EA	EA	swEA	EA	9 (5.6)	C
	1B.1.2.3 (Δ146-147)	N2-Scot	EA	EA	EA	EA	swEA	EA	5 (3.1)	C
	1B.1.2.3 (Δ146-147)	N2-Scot	EA	EA	EA	EA	swEU	EA	4 (2.5)	C
	1B.1.2.1	N2-Gent	EA	EA	EA	EA	swEU	EA	4 (2.5)	E
H1_hu_N1	1B.1.2.3	N1-EA	EA	EA	EA	EA	swEU	EA	4 (2.5)	H

* Genotype previously described by Watson et al. with the respective nomenclature [13]. Abbreviations of lineages: ‘EA’ for Eurasian avian-like, ‘pdm’ for H1N1 pandemic 2009, ‘N2-Scot’ for Scotland-like, ‘N2-Gent’ for Gent-like, ‘N2hu’ for human seasonal N2, ‘Dk-like’ for M Danish-like group, ‘swEA’ and ‘swEU’ for M swine Eurasian avian-like ‘recent’ and M swine European groups, respectively, as described in this study. Background takes up the colors used in phylogenies for the different lineages and/or clades (Figure 2, Figure 4, Figure 5, Figure 6 and Figure 7).

## Supplementary Materials

The following are available online at https://www.mdpi.com/1999-4915/12/11/1304/s1, Table S1: Genbank accession numbers for swIAV sequences isolated in France and used in this study, Table S2: List of residues identified as possibly involved in changing the pathogenicity, virulence or fitness of IAVs, Table S3: HI titers of hyperimmune antisera directed against reference H1_av_, H1_hu_, H1pdm and classical swine H1 viruses when tested to swIAV of (A) H1_av_Ny or (B) H1_hu_Ny lineage isolated in France between 2000–2018, Table S4: Specific mutation patterns identified with AVANA software which characterize deduced amino acid sequences of internal proteins from strains of the three genogroups A, B and C within HA-1C.2.1 clade, Figure S1: Detailed results of M-gene metagenomics analysis.
