# Supplementary material for: Genetic and Antigenic Evolution of European Swine Influenza A Viruses of HA-1C (Avian-Like) and HA-1B (Human-Like) Lineages in France from 2000 to 2018
Source: Viruses. 2020 Nov 13;12(11):1304. doi: 10.3390/v12111304 (PMC7697621; doi:10.3390/v12111304)

Figure 1. Genogroups identified by hierarchical clustering analysis among M-gene sequences from human and swine IAVs from North hemisphere

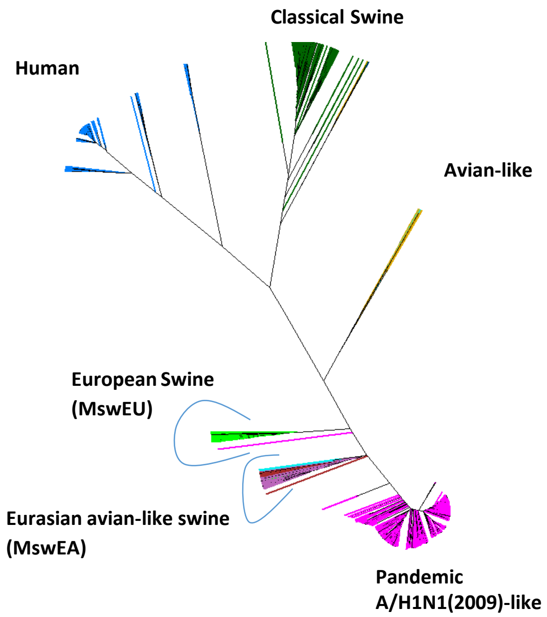

Figure 2. Distribution of M sequences from MswEA and MswEU per IAV subtypes from 1996 to 2016

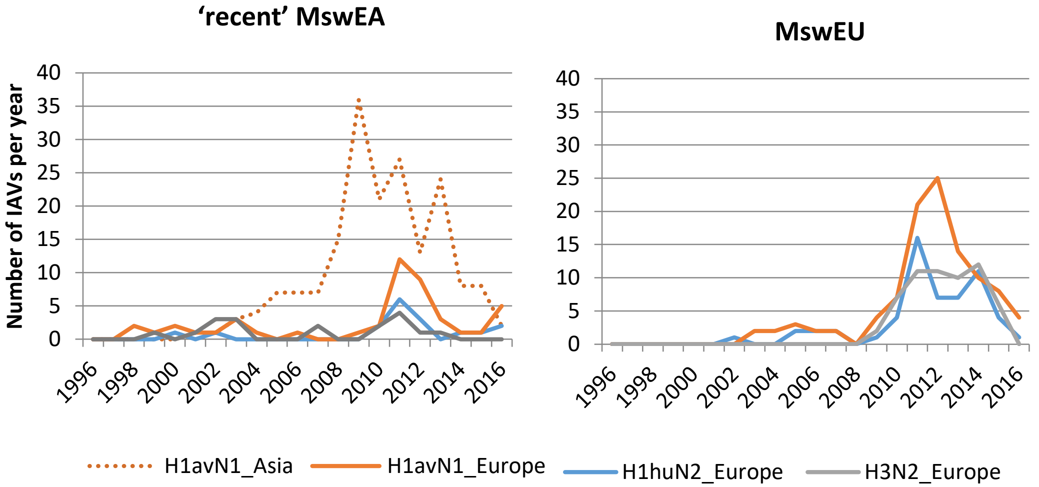

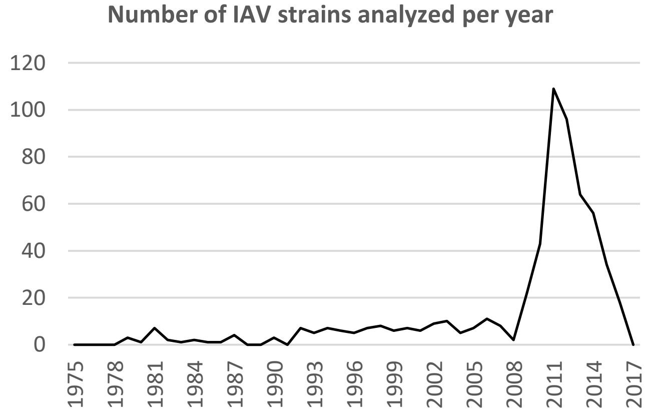

Table 1. Distribution of M sequences within the so called 'Eurasian avian-like swine' lineage grouping the subgroups 'MswEU', 'MswEA' and 'Avian-like', per host/continent and IAV subtypes

		Swine North America	Swine Asia	Swine Europe	Human Europe	H1N1	H1N2	H3N2	H5N1	H9N2	Other
MswEU		0	0	248	2	110	76	60	0	0	4
MswEA	'Ancestral' <2009	1	21	103	0	68	25	30	0	0	2
	Danish $H1_{av}N2$-like	0	3	21	0	6	15	0	0	1	2
	'Recent' >2000	0	205	102	0	247	40	18	0	0	2
Avian-like		5	51	1	3	2	1	1	20	17	19

Figure 3. Distribution of M genogroup associated to H1avNy and H1huNy strains isolated from pigs in France between 2000 and 2018

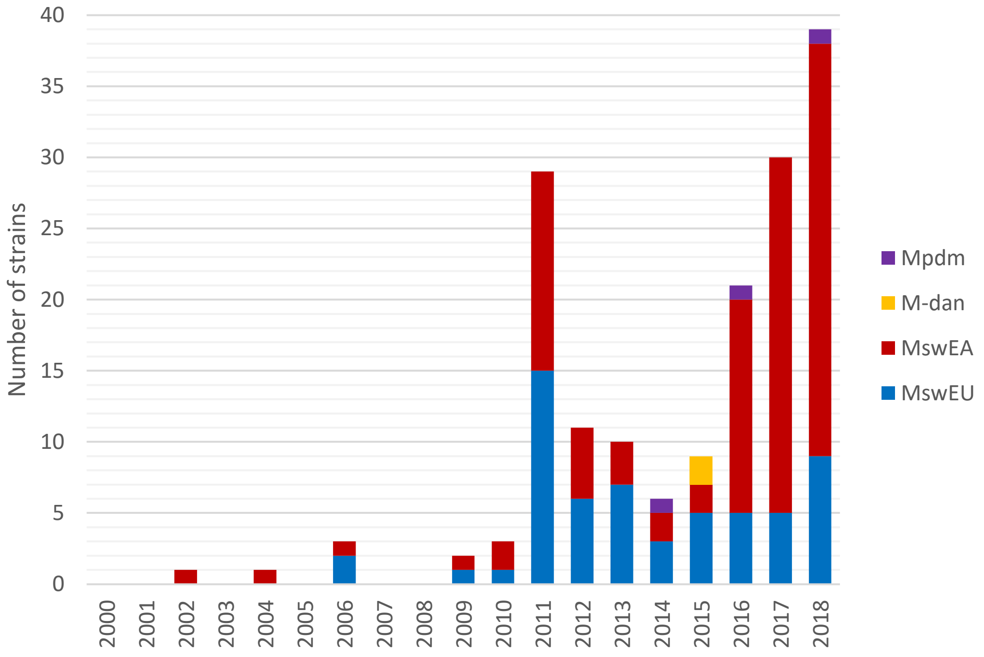

Supplement: Supplementary file 1 [file viruses-12-01304-s001.zip › Supp Fig S1_Detailled_M-gene.pdf]
