# Supplementary material for: Genetic and Antigenic Evolution of European Swine Influenza A Viruses of HA-1C (Avian-Like) and HA-1B (Human-Like) Lineages in France from 2000 to 2018
Source: Viruses. 2020 Nov 13;12(11):1304. doi: 10.3390/v12111304 (PMC7697621; doi:10.3390/v12111304)
Supplement: Supplementary file 1 [file viruses-12-01304-s001.zip › Table_S1_Genbank_acc_numbers.pdf]

Table S1. Genbank accession numbers for swIAV sequences isolated in France and used in this study

Strain name	Collection date	Subtype	HA clade (subgroup)	PB2	PB1	PA	HA	NP	NA	M	NS	References
A/swine/Marseille/2260/1980	1980	$H1_{av}N1$	1C.1	KJ889313	KJ889314	KJ889315	KJ889316	KJ889317	KJ889318	KJ889319	KJ889320	Krumbholz et al 2014
A/swine/Finnistere/2899/1982	1982	$H1_{av}N1$	1C.1	AJ311457	AJ311462	AJ311463	CY116348	CY116349	CY116350	CY116351	CY116352	Lycett et al 2012
A/swine/France/WVL3/1984	1984	$H1_{av}N1$	1C.1	CY037972	CY037973	CY037974	CY037975	CY037976	CY037977	CY037978	CY037979	Dunham et al 2009
A/swine/France/WVL4/1985	1985	$H1_{av}N1$	1C.1	CY037980	CY037981	CY037982	CY037983	CY037984	CY037985	CY037986	CY037987	Dunham et al 2009
A/swine/France/WVL8/1992	1992	$H1_{av}N1$	1C.1	CY038012	CY038013	CY038014	CY038015	CY038016	CY038017	CY038018	CY038019	Dunham et al 2009
A/swine/OMS/2111/1995	1995	$H1_{av}N1$	1C.1-2-like	CY116393	CY116394	CY116395	CY116396	CY116397	CY116398	CY116399	CY116400	Lycett et al 2012
A/swine/France/WVL13/1995	1995	$H1_{av}N1$	1C.1-2-like	CY037926	CY037927	CY037928	CY037929	CY037930	CY037931	CY037932	CY037933	Dunham et al 2009
A/swine/OMS/2112/1995	1995	$H1_{av}N1$	1C.1-2-like	CY116401	CY116402	CY116403	CY116404	CY116405	CY116406	CY116407	CY116408	Lycett et al 2012
A/swine/Cotes d'Armor/790/97	1997	$H1_{hu}N2$	1B.1.2.3	AJ306845	AJ306865	AJ311207	AJ344007	AJ307065	AJ412705	AJ316049	AJ344026	Marozin et al 2002
A/swine/Cotes d'Armor/2433/98	1998	$H1_{hu}N2$	1B.1.2.3	-na-	AJ306864	AJ311205	AJ412710	AJ307066	AJ412704	-na-	AJ344031	Marozin et al 2002
A/swine/Cotes d'Armor/1482/99	1999	$H1_{av}N1$	1C.1-2-like	-na-	-na-	-na-	AJ412711	-na-	AJ410883	-na-	-na-	Marozin et al 2002
A/swine/Ille et Vilaine/1455/1999	1999	$H1_{av}N1$	1C.2.3	CY116377	CY116378	CY116379	CY116380	CY116381	CY116382	CY116383	CY116384	Lycett et al 2012
A/swine/Cotes d'Armor/1488/99	1999	$H1_{av}N1$	1C.2.3	AJ306848	AJ306856	AJ311208	AJ412712	AJ307069	AJ410881	-na-	AJ344027	Marozin et al 2002
A/swine/Cotes d'Armor/1515/99	1999	$H1_{av}N1$	1C.1-2-like	-na-	-na-	-na-	AJ344002	-na-	AJ410884	-na-	-na-	Marozin et al 2002
A/swine/Cotes d'Armor/604/99	1999	$H1_{hu}N2$	1B.1.1	AJ306850	AJ306863	AJ312838	AJ412709	AJ307074	AJ412702	AJ316051	AJ316062	Marozin et al 2002
A/swine/Cotes d'Armor/1121/00	2000	$H1_{av}N1$	1C.2.3	-na-	-na-	-na-	AJ412708	-na-	AJ410875	-na-	-na-	Marozin et al 2002
A/swine/Cotes d'Armor/799/00	2000	$H1_{hu}N2$	1B.1.2.3	-na-	-na-	-na-	AJ344006	-na-	AJ412694	-na-	-na-	Marozin et al 2002
A/swine/Cotes d'Armor/800/00	2000	$H1_{hu}N2$	1B.1.2.3	AJ306841	AJ306859	AJ312837	AJ344005	AJ307061	AJ412695	AJ316050	AJ344039	Marozin et al 2002
A/swine/Cotes d'Armor/98574/2001	2001	$H1_{hu}N1$	1B.1.1	-na-	-na-	-na-	AM503897	-na-	AM503897	-na-	-na-	Franck et al 2007
A/swine/Cotes d'Armor/60293/2001	2001	$H1_{hu}N1$	1B.1.1	-na-	-na-	-na-	AM503896	-na-	AM777826	-na-	-na-	Franck et al 2007
A/swine/Cotes d'Armor/1624/2002	2002	$H1_{av}N1$	1C.2.3	CY116385	CY116386	CY116387	CY116388	CY116389	CY116390	CY116391	CY116392	Lycett et al 2012
A/swine/Cotes d'Armor/1190/2002	2002	$H1_{hu}N2$	1B.1.2.3	-na-	-na-	-na-	CY116372	-na-	CY116374	CY116375	-na-	Lycett et al 2012
A/swine/Cotes dArmor/43394/2002	2002	$H1_{hu}N2$	1B.1.2.3	-na-	-na-	-na-	AM503899	-na-	AM777822	-na-	-na-	This study
A/swine/Finnistere/800/2004	2004	$H1_{av}N1$	1C.2.3	CY116353	CY116354	CY116355	CY116356	CY116357	CY116358	CY116359	CY116360	Lycett et al 2012
A/swine/Cotes d'Armor/016007/2005	2005-07-12	$H1_{hu}N1$	1B.1.1	-na-	-na-	-na-	AM777810	-na-	AM777830	-na-	-na-	This study
A/swine/Cotes d'Armor/0227/2005	2005-10-17	$H1_{av}N1$	1C.2.1	-na-	-na-	-na-	AM490221	-na-	AM777828	-na-	-na-	This study
A/swine/France/CotesdArmor-0113/2006	2006-04-25	$H1_{hu}N2$	1B.1.2.3 (D)	KR701501	KR701502	KR701503	AM503902	KR701504	KR701505	KR701506	KR701507	Watson et al 2015
A/swine/Cotes d'Armor/0136_18/2006	2006-05-09	$H1_{av}N1$	1C.2.1	-na-	-na-	-na-	AM490223	-na-	AM777827	-na-	-na-	This study
A/swine/Cotes d'Armor/0136_17/2006	2006-05-09	$H1_{hu}N2$	1B.1.2.3	-na-	-na-	-na-	AM490224	-na-	AM777821	-na-	-na-	This study
A/swine/Cotes dArmor/0123/2006	2006-05-12	$H1_{hu}N2$	1B.1.2.3 (D)	-na-	-na-	-na-	AM503901	-na-	AM777820	-na-	-na-	This study

A/swine/France/Cotes d'Armor-0190/2006	2006-08-01	$H1_{hu}N1$	1B.1.2.3 *	MT379427	MT379428	MT379429	MT379430	MT379431	MT379432	MT379433	MT379434	This study
A/swine/France/Cotes d'Armor-0214/2006	2006-09-01	$H1_{hu}N2$	1B.1.1	MT394946	MT394947	MT394948	MT394949	MT394950	MT394951	MT394952	MT394953	This study
A/swine/Cotes d'Armor/002007/2006	2006-12-22	$H1_{av}N1$	1C.2.1	-na-	-na-	-na-	AM778090	-na-	AM777829	-na-	-na-	This study
A/swine/France/CotesdArmor-0041/2011	2007-02-01	$H1_{hu}N2$	1B.1.2.3	KR700213	KR700214	KR700215	KR700216	KR700217	KR700218	KR700219	KR700220	Watson et al 2015
A/swine/Cotes dArmor/0040/2007	2007-02-01	$H1_{hu}N2$	1B.1.2.3 (D)	-na-	-na-	-na-	AM503547	-na-	AM777817	-na-	-na-	This study
A/swine/Morbihan/0028/2007	2007-02-05	$H1_{hu}N2$	1B.1.2.3 (D)	-na-	-na-	-na-	AM777813	-na-	AM777816	-na-	-na-	This study
A/swine/Cotes d'Armor/0111/2007	2007-03-01	$H1_{hu}N2$	1B.1.2.3	-na-	-na-	-na-	AM777814	-na-	AM777819	-na-	-na-	This study
A/swine/Finistere/0045/2008	2008-02-12	$H1_{hu}N2$	1B.1.2.3	-na-	-na-	-na-	FN646097	-na-	FN646092	-na-	-na-	This study
A/swine/Cotes d'Armor/0102/2008	2008-04-08	$H1_{av}N2$	1C.2.1	-na-	-na-	-na-	FN646098	-na-	FN646095	-na-	-na-	This study
A/swine/Cotes d'Armor/0158/2008	2008-05-19	$H1_{av}N1$	1C.2.1	-na-	-na-	-na-	FN646101	-na-	FN646096	-na-	-na-	This study
A/swine/Cotes d'Armor/1174/2009	2009	$H1_{hu}N2$	1B.1.2.3	CY116364	CY116365	CY116366	CY116367	CY116368	CY116369	CY116370	CY116371	Lycett et al 2012
A/swine/Finistere/0028/2009	2009-02-04	$H1_{av}N1$	1C.2.1	-na-	-na-	-na-	HE577046	-na-	FR878010	-na-	-na-	This study
A/swine/Cotes d'Armor/0388/2009	2009-07-28	$H1_{av}N1$	1C.2.1	KR701495	KR701496	KR701497	KC881265	KR701498	FR871194	KR701499	KR701500	Watson et al 2015
A/swine/Aisne/0054/2010	2010	$H1_{av}N1$	1C.2.1	-na-	-na-	-na-	CY110764	-na-	CY110765	-na-	-na-	This study
A/swine/France/Cotes d'Armor-0070/2010	2010-03-19	$H1_{hu}N1$	1B.1.2.3	MT379419	MT379420	MT379421	MT379422	MT379423	MT379424	MT379425	MT379426	This study
A/swine/Cotes d'Armor/0104/2010	2010-05-21	$H1_{av}N1$	1C.2.1	-na-	-na-	-na-	CY110766	-na-	CY110767	-na-	-na-	This study
A/swine/Morbihan/0163/2010	2010-06-18	$H1_{hu}N1$	1B.1.1	-na-	-na-	-na-	KC345638	-na-	KC345652	-na-	-na-	This study
A/swine/Finistere/0181/2010	2010-08-02	$H1_{av}N2$	1C.2.1	-na-	-na-	-na-	KC345631	-na-	HE600192	-na-	-na-	This study
A/swine/Cotes d'Armor/0186/2010	2010-09-01	$H1_{av}N2$	1C.2.1	-na-	-na-	-na-	CY115832	-na-	HE577045	-na-	-na-	This study
A/swine/Finistere/0188/2010	2010-09-02	$H1_{av}N1$	1C.2.1	-na-	-na-	-na-	KC345632	-na-	HE600193	-na-	-na-	This study
A/swine/Ile et Vilaine/0225/2010	2010-09-30	$H1_{hu}N2$	1B.1.2.3	-na-	-na-	-na-	KC345633	-na-	KC345634	-na-	-na-	This study
A/swine/France/CotesdArmor-0275/2010	2010-11-23	$H1_{av}N1$	1C.2.1	KR699731	-na-	KR699733	KR699734	KR699735	KR699736	KR699737	KR699738	Watson et al 2015
A/swine/France/56-110525/2010	2010-11-26	$H1_{hu}N1$	1B.1.2.3 (D)	KR700514	KR700515	KR700516	KR700517	KR700518	KR700519	KR700520	KR700521	Watson et al 2015
A/swine/France/CotesdArmor-0023/2011	2011-01-01	$H1_{av}N1$	1C.2.1	KR700269	-na-	KR700270	KR700271	KR700272	KR700273	KR700274	KR700275	Watson et al 2015
A/swine/France/CotesdArmor-0041/2011	2011-01-01	$H1_{hu}N2$	1B.1.2.3 (D)	KR700213	KR700214	KR700215	KR700216	KR700217	KR700218	KR700219	KR700220	Watson et al 2015
A/swine/France/Finistere-0011/2011	2011-01-11	$H1_{av}N1$	1C.2.1	KR699739	KR699740	KR699741	KR699742	KR699743	KR699744	KR699745	KR699746	Watson et al 2015
A/swine/France/CotesdArmor-0025/2011	2011-01-20	$H1_{hu}N2$	1B.1.2.3 (D)	KR700206	MT394954	KR700207	KR700208	KR700209	KR700210	KR700211	KR700212	Watson et al 2015
A/swine/France/Ille et Vilaine-0115/2011	2011-02-01	$H1_{av}N1$	1C.2.1	MT394979	MT394980	MT394981	MT394982	MT394983	MT394984	MT394985	MT394986	This study
A/swine/France/CotesdArmor-0105/2011	2011-02-01	$H1_{hu}N2$	1B.1.2.3	KR700221	KR700222	KR700223	KR700224	KR700225	KR700226	KR700227	KR700228	Watson et al 2015
A/swine/France/CotesdArmor-0074/2011	2011-02-01	$H1_{hu}N2$	1B.1.2.3 (D)	KR700237	KR700238	KR700239	KR700240	KR700241	KR700242	KR700243	KR700244	Watson et al 2015
A/swine/France/22-110529/2011	2011-02-08	$H1_{av}N2$	1C.2.1	KR700323	KR700324	KR700325	KR700326	-na-	KR700328	KR700329	KR700330	Watson et al 2015
A/swine/France/CotesdArmor-0147/2011	2011-04-05	$H1_{av}N1$	1C.2.1	KR700253	KR700254	KR700255	KR700256	KR700257	KR700258	KR700259	KR700260	Watson et al 2015
A/swine/France/IIIeetVilaine-0187/2011	2011-04-07	$H1_{av}N1$	1C.2.1	KR699747	KR699748	KR699749	KR699750	KR699751	KR699752	KR699753	KR699754	Watson et al 2015
A/swine/France/CotesdArmor-0198/2011	2011-04-11	$H1_{hu}N2$	1B.1.2.3	KR700190	KR700191	KR700192	KR700193	KR700194	KR700195	KR700196	KR700197	Watson et al 2015

A/swine/France/Finistere-0186/2011	2011-04-14	$H1_{hu}N2$	1B.1.2.3	MT394971	MT394972	MT394973	MT394974	MT394975	MT394976	MT394977	MT394978	This study
A/swine/France/Morbihan-0213/2011	2011-04-18	$H1_{av}N2$	1C.2.1	KR699755	KR699756	KR699757	KR699758	KR699759	KR699760	KR699761	KR699762	Watson et al 2015
A/swine/France/IIleetVilaine-0266/2011	2011-05-24	$H1_{av}N1$	1C.2.1	KR700229	KR700230	KR700231	KR700232	KR700233	KR700234	KR700235	KR700236	Watson et al 2015
A/swine/France/Morbihan-0285/2011	2011-05-30	$H1_{av}N1$	1C.2.1	KR700245	KR700246	KR700247	KR700248	KR700249	KR700250	KR700251	KR700252	Watson et al 2015
A/swine/France/IIleetVilaine-0346/2011	2011-06-01	$H1_{hu}N2$	1B.1.2.3 (D)	KR701483	KR701484	KR701485	KC894804	KR701486	KC894807	KR701487	KR701488	Watson et al 2015
A/swine/France/Cotes d'Armor-0295/2011	2011-06-06	$H1_{hu}N2$	1B.1.2.3 (D)	MT394955	MT394956	MT394957	MT394958	MT394959	MT394960	MT394961	MT394962	This study
A/swine/France/CotesdArmor-0282/2011	2011-06-06	$H1_{av}N1$	1C.2.1	KR699803	KR699804	KR699805	KR699806	KR699807	MT394952	KR699808	KR699809	Watson et al 2015
A/swine/France/Morbihan-0294/2011	2011-06-14	$H1_{av}N1$	1C.2.1	KR699763	KR699764	KR699765	KR699766	KR699767	KR699768	KR699769	KR699770	Watson et al 2015
A/swine/France/Vendee-0301/2011	2011-06-14	$H1_{av}N1$	1C.2.1	KR699771	KR699772	KR699773	KR699774	KR699775	KR699776	KR699777	KR699778	Watson et al 2015
A/swine/France/CotesdArmor-0292/2011	2011-06-15	$H1_{av}N1$	1C.2.1	KR700261	KR700262	KR700263	KR700264	KR700265	KR700266	KR700267	KR700268	Watson et al 2015
A/swine/France/29-110307/2011	2011-06-23	$H1_{av}N1$	1C.2.1	-na-	-na-	-na-	KC894805	-na-	KC894808	-na-	-na-	This study
A/swine/France/IIleetVilaine-0404/2011	2011-06-28	$H1_{hu}N2$	1B.1.2.3	KR700276	KR700277	KR700278	KR700279	KR700280	KR700281	KR700282	KR700283	Watson et al 2015
A/swine/France/IIleetVilaine-0354/2011	2011-06-30	$H1_{av}N1$	1C.2.1	KR700198	KR700199	KR700200	KR700201	-na-	KR700203	KR700204	KR700205	Watson et al 2015
A/swine/France/LoireAtlantique-0405/2011	2011-07-01	$H1_{av}N1$	1C.2.1	KR699779	KR699780	KR699781	KR699782	KR699783	KR699784	KR699785	KR699786	Watson et al 2015
A/swine/France/CotesdArmor-0364/2011	2011-07-05	$H1_{av}N1$	1C.2.1	KR700904	-na-	KR700905	KR700906	KR700907	KR700908	KR700909	KR700910	Watson et al 2015
A/swine/France/IIleetVilaine-0415/2011	2011-08-01	$H1_{hu}N2$	1B.1.2.3	KR699787	KR699788	KR699789	KR699790	KR699791	KR699792	KR699793	KR699794	Watson et al 2015
A/swine/France/Cotes d'Armor-0455/2011	2011-09-05	$H1_{av}N1$	1C.2.1	MT394963	MT394964	MT394965	MT394966	MT394967	MT394968	MT394969	MT394970	This study
A/swine/France/CotesdArmor-0558/2011	2011-09-14	$H1_{hu}N2$	1B.1.2.3 (D)	KR700315	KR700316	KR700317	KR700318	KR700319	KR700320	KR700321	KR700322	Watson et al 2015
A/swine/France/Morbihan-0599/2011	2011-10-27	$H1_{av}N2$	1C.2.1	KR700292	MT394953	KR700293	KR700294	KR700295	KR700296	KR700297	KR700298	Watson et al 2015
A/swine/France/Ille et Vilaine-0648/2011	2011-11-07	$H1_{hu}N2$	1B.1.2.3	MT394987	MT394988	MT394989	MT394990	MT394991	MT394992	MT394993	MT394994	This study
A/swine/France/Morbihan-0617/2011	2011-11-14	$H1_{av}N1$	1C.2.1	MT394940	-na-	MT394941	MT394942	MT394943	-na-	MT394944	MT394945	This study
A/swine/France/41-120137/2012	2012-01-10	$H1_{av}N1$	1C.2.1	MT379003	MT379004	MT379005	MT379006	MT379007	MT379008	MT379009	MT379010	This study
A/swine/France/35-120024/2012	2012-01-19	$H1_{hu}N2$	1B.1.2.3	KR700307	KR700308	KR700309	KR700310	KR700311	KR700312	KR700313	KR700314	Watson et al 2015
A/swine/France/22-120067/2012	2012-02-08	$H1_{hu}N1$	1B.1.2.3 **	KR700967	KR700968	KR700969	KR700970	KR700971	KR700972	KR700973	KR700974	Watson et al 2015
A/swine/France/29-120064/2012	2012-02-09	$H1_{av}N1$	1C.2.1	KR700284	KR700285	KR700286	KR700287	KR700288	KR700289	KR700290	KR700291	Watson et al 2015
A/swine/France/22-120146/2012	2012-04-17	$H1_{av}N1$	1C.2.1	KR700911	KR700912	KR700913	KR700914	KR700915	KR700916	KR700917	KR700918	Watson et al 2015
A/swine/France/56-120177/2012	2012-05-21	$H1_{hu}N2$	1B.1.2.3	KR701566	KR701567	KR701568	KC295234	KR701569	KR701570	KR701571	KR701572	Watson et al 2015
A/swine/France/22-120228/2012	2012-07-09	$H1_{hu}N2$	1B.1.2.3	KR700943	KR700944	KR700945	KR700946	KR700947	KR700948	KR700949	KR700950	Watson et al 2015
A/swine/France/29-120326/2012	2012-08-28	$H1_{av}N1$	1C.2.1	KR700935	KR700936	KR700937	KR700938	KR700939	KR700940	KR700941	KR700942	Watson et al 2015
A/swine/France/22-120340/2012	2012-10-09	$H1_{av}N1$	1C.2.1	KR700927	KR700928	KR700929	KR700930	KR700931	KR700932	KR700933	KR700934	Watson et al 2015
A/swine/France/50-120350/2012	2012-10-10	$H1_{av}N1$	1C.2.1	KR700919	KR700920	KR700921	KR700922	KR700923	KR700924	KR700925	KR700926	Watson et al 2015
A/swine/France/37-120345/2012	2012-10-18	$H1_{av}N2$	1C.2.1	KR700959	KR700960	KR700961	KR700962	KR700963	KR700964	KR700965	KR700966	Watson et al 2015; Chastagner et al 2019
A/swine/France/22-120421/2012	2012-11-27	$H1_{av}N1$	1C.2.1	-na-	-na-	-na-	KM267920	-na-	KP822957	-na-	-na-	This study

A/swine/France/22-120460/2012	2012-12-19	$H1_{av}N1$	1C.2.1	-na-	-na-	-na-	KM267921	-na-	KP822958	-na-	-na-	This study
A/swine/France/35-130023/2013	2013-01-02	$H1_{hu}N2$	1B.1.2.3 (Δ146-147)	KR700983	-na-	KR700984	KR700985	KR700986	KR700987	KR700988	KR700989	Watson et al 2015
A/swine/France/01-130054/2013	2013-01-22	$H1_{av}N1$	1C.2.2	KR700975	KR700976	KR700977	KR700978	KR700979	KR700980	KR700981	KR700982	Watson et al 2015
A/swine/France/53-130065/2013	2013-01-23	$H1_{av}N1$	1C.2.1	MT394924	MT394925	MT394926	MT394927	MT394928	MT394929	MT394930	MT394931	This study
A/swine/France/70-130144/2013	2013-03-27	$H1_{av}N1$	1C.2.1	MT379355	MT379356	MT379357	MT379358	MT379359	MT379360	MT379361	MT379362	This study
A/swine/France/01-130145/2013	2013-04-09	$H1_{av}N1$	1C.2.2	MT378595	MT378596	MT378597	MT378598	MT378599	MT378600	MT378601	MT378602	This study
A/swine/France/59-130174/2013	2013-04-15	$H1_{av}N1$	1C.2.1	MT379195	MT379196	MT379197	MT379198	MT379199	MT379200	MT379201	MT379202	This study
A/swine/France/22-130212/2013	2013-06-05	$H1_{hu}N2$	1B.1.2.3 (Δ146-147)	KM267908	KM267909	KM267910	KJ128323	KM267911	KJ128301	KM267912	KM267913	This study
A/swine/France/50-130337/2013	2013-09-17	$H1_{av}N1$	1C.2.1	MT379043	MT379044	MT379045	MT379046	MT379047	MT379048	MT379049	MT379050	This study
A/swine/France/29-130398/2013	2013-10-24	$H1_{av}N1$	1C.2.1 (A)	MT378571	MT378572	MT378573	MT378574	MT378575	MT378576	MT378577	MT378578	This study
A/swine/France/64-130454/2013	2013-12-19	$H1_{av}N1$	1C.2.1	MT379323	MT379324	MT379325	MT379326	MT379327	MT379328	MT379329	MT379330	This study
A/swine/France/01-140489/2014	2014	$H1_{av}N1$	1C.2.1 (B)	MT378563	MT378564	MT378565	MT378566	MT378567	MT378568	MT378569	MT378570	This study
A/swine/France/35-140041/2014	2014-01-14	$H1_{hu}N2$	1B.1.2.3 (Δ146-147)	-na-	-na-	-na-	KM267933	-na-	KY241123	-na-	-na-	This study
A/swine/France/50-140056/2014	2014-01-27	$H1_{hu}N2$	1B.1.2.3 (Δ146-147)	MT379051	MT379052	MT379053	MT379054	MT379055	MT379056	MT379057	MT379058	This study
A/swine/France/50-140058/2014	2014-01-28	$H1_{hu}N2$	1B.1.2.3 (Δ146-147)	-na-	-na-	-na-	KY241126	-na-	KY241127	-na-	-na-	This study
A/swine/France/22-140062/2014	2014-02-04	$H1_{hu}N2$	1B.1.2.3 (Δ146-147)	-na-	-na-	-na-	KY241128	-na-	KY241129	-na-	-na-	This study
A/swine/France/22-140075/2014	2014-02-20	$H1_{hu}N2$	1B.1.2.3 (Δ146-147)	-na-	-na-	-na-	KY241130	-na-	KY241131	-na-	-na-	This study
A/swine/France/22-140102/2014	2014-02-28	$H1_{hu}N2$	1B.1.2.3 (Δ146-147)	-na-	-na-	-na-	KY241132	-na-	KY241133	-na-	-na-	This study
A/swine/France/79-140150/2014	2014-03-11	$H1_{av}N1$	1C.2.1	MT379387	MT379388	MT379389	MT379390	MT379391	MT379392	MT379393	MT379394	This study
A/swine/France/29-140221/2014	2014-04-17	$H1_{hu}N2$	1B.1.2.3 (Δ146-147)	-na-	-na-	-na-	KY241134	-na-	KY241135	-na-	-na-	This study
A/swine/France/22-140256/2014	2014-05-07	$H1_{hu}N2$	1B.1.2.3 (Δ146-147)	-na-	-na-	-na-	KY241136	-na-	KY241137	-na-	-na-	This study
A/swine/France/61-140350/2014	2014-06-04	$H1_{av}N2$	1C.2.1	MT379291	MT379292	MT379293	MT379294	MT379295	MT379296	MT379297	MT379298	Chastagner et al 2019
A/swine/France/29-140342/2014	2014-06-05	$H1_{hu}N2$	1B.1.2.3	MT378787	MT378788	MT378789	MT378790	MT378791	MT378792	MT378793	MT378794	This study
A/swine/France/64-140385/2014	2014-07-07	$H1_{av}N1$	1C.2.1	MT379331	MT379332	MT379333	MT379334	MT379335	MT379336	MT379337	MT379338	This study
A/swine/France/56-140486/2014	2014-11-24	$H1_{hu}N2$	1B.1.2.3 (Δ146-147)	-na-	-na-	-na-	KY241142	-na-	KY2411413	-na-	-na-	This study
A/swine/France/22-140494/2014	2014-12-03	$H1_{hu}N2$	1B.1.2.3 (Δ146-147)	-na-	-na-	-na-	KY241144	-na-	KY241145	-na-	-na-	This study
A/swine/France/22-140496/2014	2014-12-08	$H1_{hu}N2$	1B.1.2.3 (Δ146-147)	-na-	-na-	-na-	KY241146	-na-	KY241147	-na-	-na-	This study
A/swine/France/22-140510/2014	2014-12-16	$H1_{hu}N2$	1B.1.2.3 (Δ146-147)	-na-	-na-	-na-	KY241148	-na-	KY241149	-na-	-na-	This study
A/swine/France/89-150069/2015	2015-02-09	$H1_{av}N1$	1C.2.2	MT379411	MT379412	MT379413	MT379414	MT379415	MT379416	MT379417	MT379418	This study
A/swine/France/56-150097/2015	2015-03-04	$H1_{hu}N2$	1B.1.2.3 (Δ146-147)	-na-	-na-	-na-	KY241154	-na-	KU289597	-na-	-na-	This study
A/swine/France/64-150152/2015	2015-03-23	$H1_{av}N2$	1C.2	MT378579	MT378580	MT378581	MT378582	MT378583	MT378584	MT378585	MT378586	This study
A/swine/France/44-150191/2015	2015-04-28	$H1_{av}N1$	1C.2.1	-na-	-na-	-na-	KY241163	-na-	KU289594	-na-	-na-	This study
A/swine/France/05-150198/2015	2015-04-29	$H1_{av}N1$	1C.2.1 (B)	MT378627	MT378628	MT378629	MT378630	MT378631	MT378632	MT378633	MT378634	This study
A/swine/France/61-150219/2015	2015-06-05	$H1_{av}N2$	1C.2.1	MT379299	MT379300	MT379301	MT379302	MT379303	MT379304	MT379305	MT379306	This study

A/swine/France/65-150242/2015	2015-06-11	$H1_{av}N2$	1C.2	MT379339	MT379340	MT379341	MT379342	MT379343	MT379344	MT379345	MT379346	This study
A/swine/France/22-150235/2015	2015-06-19	$H1_{hu}N2$	1B.1.2.3 (Δ146-147)	-na-	-na-	-na-	KY241155	-na-	KU289591	-na-	-na-	This study
A/swine/France/53-150263/2015	2015-07-10	$H1_{av}N1$	1C.2.1 (A)	-na-	-na-	-na-	KY241166	-na-	KU289595	-na-	-na-	This study
A/swine/France/35-150267/2015	2015-07-16	$H1_{av}N1$	1C.2.1 (A)	-na-	-na-	-na-	KY241167	-na-	KU289593	-na-	-na-	This study
A/swine/France/22-150284/2015	2015-08-10	$H1_{hu}N2$	1B.1.2.3 (Δ146-147)	-na-	-na-	-na-	KY241157	-na-	KY241158	-na-	-na-	This study
A/swine/France/56-150300/2015	2015-08-17	$H1_{av}N1$	1C.2.1	-na-	-na-	-na-	KY241105	-na-	KY241106	-na-	-na-	This study
A/swine/France/62-150308/2015	2015-08-27	$H1_{av}N1$	1C.2.1 (C)	MT379307	MT379308	MT379309	MT379310	MT379311	MT379312	MT379313	MT379314	This study
A/swine/France/53-150320/2015	2015-09-07	$H1_{av}N2$	1C.2.1	-na-	-na-	-na-	KY241159	-na-	KU289596	-na-	-na-	This study
A/swine/France/72-150321/2015	2015-09-15	$H1_{av}N2$	1C.2.1	MT379363	MT379364	MT379365	MT379366	MT379367	MT379368	MT379369	MT379370	This study
A/swine/France/29-150341/2015	2015-10-06	$H1_{av}N1$	1C.2.1	MT378795	MT378796	MT378797	MT378798	MT378799	MT378800	MT378801	MT378802	This study
A/swine/France/22-150369/2015	2015-10-27	$H1_{hu}N2$	1B.1.2.3	-na-	-na-	-na-	KY241108	-na-	KY241109	-na-	-na-	This study
A/swine/France/22-150368/2015	2015-10-28	$H1_{av}N1$	1C.2.1 (A)	-na-	-na-	-na-	KY241095	-na-	KY241096	-na-	-na-	This study
A/swine/France/22-150384/2015	2015-11-02	$H1_{hu}N2$	1B.1.2.3	MT378659	MT378660	MT378661	MT378662	MT378663	MT378664	MT378665	MT378666	This study
A/swine/France/29-150396/2015	2015-11-05	$H1_{av}N1$	1C.2.1	-na-	-na-	-na-	KY241097	-na-	KY241098	-na-	-na-	This study
A/swine/France/35-150447/2015	2015-12-07	$H1_{av}N1$	1C.2.1 (A)	-na-	-na-	-na-	KY241112	-na-	KY241113	-na-	-na-	This study
A/swine/France/22-160079/2016	2016-01-27	$H1_{av}N1$	1C.2.1 (A)	KY364117	KY364118	KY364119	KY364120	KY364121	KY364122	KY364123	KY364124	This study
A/swine/France/29-160096/2016	2016-01-28	$H1_{hu}N2$	1B.1.2.3	KY364125	KY364126	KY364127	KY364128	KY364129	KY364130	KY364131	KY364132	This study
A/swine/France/59-160078/2016	2016-01-28	$H1_{av}N2$	1C.2.1 (C)	-na-	-na-	-na-	KY241161	-na-	KY241162	-na-	-na-	This study
A/swine/France/01-160099/2016	2016-02-03	$H1_{av}N1$	1C.2.1 (B)	KY364109	KY364110	KY364111	KY364112	KY364113	KY364114	KY364115	KY364116	This study
A/swine/France/35-160126/2016	2016-02-25	$H1_{av}N1$	1C.2.1	KY364133	KY364134	KY364135	KY364136	KY364137	KY364138	KY364139	KY364140	This study
A/swine/France/72-160154/2016	2016-03-24	$H1_{av}N1$	1C.2.1 (A)	KY364157	KY364158	KY364159	KY364160	KY364161	KY364162	KY364163	KY364164	This study
A/swine/France/22-160150/2016	2016-03-24	$H1_{hu}N2$	1B.1.2.3	KY364149	KY364150	KY364151	KY364152	KY364153	KY364154	KY364155	KY364156	This study
A/swine/France/37-160178/2016	2016-05-02	$H1_{av}N2$	1C.2.1	KY364173	KY364174	KY364175	KY364176	KY364177	KY364178	KY364179	KY364180	Chastagner et al 2019
A/swine/France/35-160197/2016	2016-05-18	$H1_{av}N1$	1C.2.1 (A)	MT378931	MT378932	MT378933	MT378934	MT378935	MT378936	MT378937	MT378938	This study
A/swine/France/35-160204/2016	2016-05-23	$H1_{hu}N2$	1B.1.2.3 (Δ146-147)	MT378939	MT378940	MT378941	MT378942	MT378943	MT378944	MT378945	MT378946	This study
A/swine/France/59-160211/2016	2016-05-26	$H1_{hu}N2$	1B.1.2.1	MT379203	MT379204	MT379205	MT379206	MT379207	MT379208	MT379209	MT379210	This study
A/swine/France/29-160215/2016	2016-06-07	$H1_{av}N1$	1C.2.1	MT394932	MT394933	MT394934	MT394935	MT394936	MT394937	MT394938	MT394939	This study
A/swine/France/62-160229/2016	2016-06-13	$H1_{hu}N2$	1B.1.2.1	MT379315	MT379316	MT379317	MT379318	MT379319	MT379320	MT379321	MT379322	This study
A/swine/France/29-160250/2016	2016-06-23	$H1_{av}N1$	1C.2.1 (A)	MT378803	MT378804	MT378805	MT378806	MT378807	MT378808	MT378809	MT378810	This study
A/swine/France/29-160311/2016	2016-09-01	$H1_{av}N1$	1C.2.1	MT378811	MT378812	MT378813	MT378814	MT378815	MT378816	MT378817	MT378818	This study
A/swine/France/59-160320/2016	2016-09-19	$H1_{hu}N2$	1B.1.2.1	MT379211	MT379212	MT379213	MT379214	MT379215	MT379216	MT379217	MT379218	This study
A/swine/France/69-160337/2016	2016-09-28	$H1_{av}N1$	1C.2.1 (B)	MT379347	MT379348	MT379349	MT379350	MT379351	MT379352	MT379353	MT379354	This study
A/swine/France/29-160352/2016	2016-09-28	$H1_{av}N1$	1C.2.1	MT378819	MT378820	MT378821	MT378822	MT378823	MT378824	MT378825	MT378826	This study
A/swine/France/50-160358/2016	2016-10-12	$H1_{hu}N2$	1B.1.2.3 (Δ146-147)	MT379059	MT379060	MT379061	MT379062	MT379063	MT379064	MT379065	MT379066	This study

A/swine/France/53-160350/2016	2016-10-12	$H1_{av}N1$	1C.2.1 (A)	MT379083	MT379084	MT379085	MT379086	MT379087	MT379088	MT379089	MT379090	This study
A/swine/France/29-160400/2016	2016-11-15	$H1_{hu}N2$	1B.1.2.3	MT378827	MT378828	MT378829	MT378830	MT378831	MT378832	MT378833	MT378834	This study
A/swine/France/35-160395/2016	2016-11-29	$H1_{av}N1$	1C.2.1	MT378947	MT378948	MT378949	MT378950	MT378951	MT378952	MT378953	MT378954	This study
A/swine/France/29-170332/2017	2017	$H1_{hu}N2$	1B.1.2.3	MT378891	MT378892	MT378893	MT378894	MT378895	MT378896	MT378897	MT378898	This study
A/swine/France/29-170039/2017	2017-01-19	$H1_{hu}N2$	1B.1.2.3	MT378835	MT378836	MT378837	MT378838	MT378839	MT378840	MT378841	MT378842	This study
A/swine/France/59-170028/2017	2017-01-20	$H1_{av}N1$	1C.2.1 (C)	MT379219	MT379220	MT379221	MT379222	MT379223	MT379224	MT379225	MT379226	This study
A/swine/France/22-170027/2017	2017-01-24	$H1_{av}N1$	1C.2.1	MT378667	MT378668	MT378669	MT378670	MT378671	MT378672	MT378673	MT378674	This study
A/swine/France/59-170058/2017	2017-02-13	$H1_{hu}N2$	1B.1.2.1	MT379227	MT379228	MT379229	MT379230	MT379231	MT379232	MT379233	MT379234	This study
A/swine/France/01-170070/2017	2017-02-27	$H1_{av}N1$	1C.2.1 (B)	MT378603	MT378604	MT378605	MT378606	MT378607	MT378608	MT378609	MT378610	This study
A/swine/France/56-170084/2017	2017-03-14	$H1_{av}N1$	1C.2.1 (A)	MT379107	MT379108	MT379109	MT379110	MT379111	MT379112	MT379113	MT379114	This study
A/swine/France/22-170089/2017	2017-03-22	$H1_{av}N1$	1C.2.1 (A)	MT378675	MT378676	MT378677	MT378678	MT378679	MT378680	MT378681	MT378682	This study
A/swine/France/29-170091/2017	2017-03-22	$H1_{hu}N2$	1B.1.2.3	MT378843	MT378844	MT378845	MT378846	MT378847	MT378848	MT378849	MT378850	This study
A/swine/France/29-170114/2017	2017-04-03	$H1_{av}N1$	1C.2.1 (A)	MT378851	MT378852	MT378853	MT378854	MT378855	MT378856	MT378857	MT378858	This study
A/swine/France/59-170121/2017	2017-04-25	$H1_{av}N1$	1C.2.1 (C)	MT379235	MT379236	MT379237	MT379238	MT379239	MT379240	MT379241	MT379242	This study
A/swine/France/53-170144/2017	2017-05-15	$H1_{av}N1$	1C.2.1	MT379091	MT379092	MT379093	MT379094	MT379095	MT379096	MT379097	MT379098	This study
A/swine/France/56-170149/2017	2017-05-22	$H1_{av}N1$	1C.2.1 (A)	MT379115	MT379116	MT379117	MT379118	MT379119	MT379120	MT379121	MT379122	This study
A/swine/France/56-170177/2017	2017-05-24	$H1_{av}N1$	1C.2.1	MT379131	MT379132	MT379133	MT379134	MT379135	MT379136	MT379137	MT379138	This study
A/swine/France/24-170162/2017	2017-06-01	$H1_{av}N1$	1C.2.1 (A)	MT378779	MT378780	MT378781	MT378782	MT378783	MT378784	MT378785	MT378786	This study
A/swine/France/56-170170/2017	2017-06-09	$H1_{hu}N2$	1B.1.2.3 (Δ146-147)	MT379123	MT379124	MT379125	MT379126	MT379127	MT379128	MT379129	MT379130	This study
A/swine/France/29-170184/2017	2017-07-18	$H1_{hu}N2$	1B.1.2.3	MT378859	MT378860	MT378861	MT378862	MT378863	MT378864	MT378865	MT378866	This study
A/swine/France/29-170198/2017	2017-07-26	$H1_{hu}N2$	1B.1.2.3	MT378875	MT378876	MT378877	MT378878	MT378879	MT378880	MT378881	MT378882	This study
A/swine/France/29-170197/2017	2017-08-02	$H1_{av}N1$	1C.2.1	MT378867	MT378868	MT378869	MT378870	MT378871	MT378872	MT378873	MT378874	This study
A/swine/France/22-170192/2017	2017-08-09	$H1_{av}N1$	1C.2.1 (A)	MT378683	MT378684	MT378685	MT378686	MT378687	MT378688	MT378689	MT378690	This study
A/swine/France/56-170226/2017	2017-08-30	$H1_{av}N1$	1C.2.1	MT379139	MT379140	MT379141	MT379142	MT379143	MT379144	MT379145	MT379146	This study
A/swine/France/35-170233/2017	2017-09-11	$H1_{av}N1$	1C.2.1 (A)	MT378955	MT378956	MT378957	MT378958	MT378959	MT378960	MT378961	MT378962	This study
A/swine/France/59-170241/2017	2017-09-15	$H1_{av}N1$	1C.2.1 (C)	MT379243	MT379244	MT379245	MT379246	MT379247	MT379248	MT379249	MT379250	This study
A/swine/France/35-170242/2017	2017-09-27	$H1_{hu}N2$	1B.1.2.3 (Δ146-147)	MT378963	MT378964	MT378965	MT378966	MT378967	MT378968	MT378969	MT378970	This study
A/swine/France/56-170294/2017	2017-11-13	$H1_{av}N1$	1C.2.1	MT379147	MT379148	MT379149	MT379150	MT379151	MT379152	MT379153	MT379154	This study
A/swine/France/22-170307/2017	2017-11-20	$H1_{av}N1$	1C.2.1	MT378691	MT378692	MT378693	MT378694	MT378695	MT378696	MT378697	MT378698	This study
A/swine/France/29-170306/2017	2017-11-21	$H1_{av}N1$	1C.2.1 (A)	MT378883	MT378884	MT378885	MT378886	MT378887	MT378888	MT378889	MT378890	This study
A/swine/France/50-170323/2017	2017-11-23	$H1_{av}N1$	1C.2.1 (A)	MT379067	MT379068	MT379069	MT379070	MT379071	MT379072	MT379073	MT379074	This study
A/swine/France/22-170335/2017	2017-12-14	$H1_{hu}N2$	1B.1.2.3	MT378699	MT378700	MT378701	MT378702	MT378703	MT378704	MT378705	MT378706	This study
A/swine/France/59-170345/2018	2017-12-19	$H1_{av}N1$	1C.2.1 (C)	MT379251	MT379252	MT379253	MT379254	MT379255	MT379256	MT379257	MT379258	This study
A/swine/France/50-170342/2017	2017-12-20	$H1_{hu}N2$	1B.1.2.3 (Δ146-147)	MT379075	MT379076	MT379077	MT379078	MT379079	MT379080	MT379081	MT379082	This study

A/swine/France/22-180018/2018	2018-01-02	$H1_{av}N1$	1C.2.1	MT378723	MT378724	MT378725	MT378726	MT378727	MT378728	MT378729	MT378730	This study
A/swine/France/22-180008/2018	2018-01-03	$H1_{av}N1$	1C.2.1	MT378707	MT378708	MT378709	MT378710	MT378711	MT378712	MT378713	MT378714	This study
A/swine/France/22-180017/2018	2018-01-11	$H1_{av}N1$	1C.2.1 (A)	MT378715	MT378716	MT378717	MT378718	MT378719	MT378720	MT378721	MT378722	This study
A/swine/France/29-180023/2018	2018-01-12	$H1_{hu}N2$	1B.1.2.3 **	MT378899	MT378900	MT378901	MT378902	MT378903	MT378904	MT378905	MT378906	This study
A/swine/France/01-180027/2018	2018-01-17	$H1_{av}N1$	1C.2.1 (B)	MT378587	MT378588	MT378589	MT378590	MT378591	MT378592	MT378593	MT378594	This study
A/swine/France/29-180035/2018	2018-01-24	$H1_{av}N1$	1C.2.1 (A)	MT378907	MT378908	MT378909	MT378910	MT378911	MT378912	MT378913	MT378914	This study
A/swine/France/72-180053/2018	2018-01-29	$H1_{av}N1$	1C.2.1 (A)	MT379371	MT379372	MT379373	MT379374	MT379375	MT379376	MT379377	MT379378	This study
A/swine/France/22-180048/2018	2018-01-29	$H1_{hu}N2$	1B.1.2.3	MT378731	MT378732	MT378733	MT378734	MT378735	MT378736	MT378737	MT378738	This study
A/swine/France/59-180117/2018	2018-02-13	$H1_{av}N1$	1C.2.1 (C)	MT379259	MT379260	MT379261	MT379262	MT379263	MT379264	MT379265	MT379266	This study
A/swine/France/29-180124/2018	2018-02-26	$H1_{hu}N2$	1B.1.2.3 (Δ146-147)	MT378915	MT378916	MT378917	MT378918	MT378919	MT378920	MT378921	MT378922	This study
A/swine/France/29-180140/2018	2018-03-13	$H1_{hu}N2$	1B.1.2.3	MT378995	MT378996	MT378997	MT378998	MT378999	MT379000	MT379001	MT379002	This study
A/swine/France/22-180175/2018	2018-03-23	$H1_{hu}N2$	1B.1.2.3	MT378739	MT378740	MT378741	MT378742	MT378743	MT378744	MT378745	MT378746	This study
A/swine/France/29-180202/2018	2018-04-08	$H1_{av}N1$	1C.2.1	MT378923	MT378924	MT378925	MT378926	MT378927	MT378928	MT378929	MT378930	This study
A/swine/France/01-180255/2018	2018-05-02	$H1_{av}N1$	1C.2.1 (B)	MT378611	MT378612	MT378613	MT378614	MT378615	MT378616	MT378617	MT378618	This study
A/swine/France/22-180254/2018	2018-05-14	$H1_{hu}N2$	1B.1.2.3	MT378747	MT378748	MT378749	MT378750	MT378751	MT378752	MT378753	MT378754	This study
A/swine/France/01-180274/2018	2018-05-16	$H1_{av}N1$	1C.2.1 (B)	MT378619	MT378620	MT378621	MT378622	MT378623	MT378624	MT378625	MT378626	This study
A/swine/France/22-180287/2018	2018-06-06	$H1_{hu}N2$	1B.1.2.3	MT378755	MT378756	MT378757	MT378758	MT378759	MT378760	MT378761	MT378762	This study
A/swine/France/35-180318/2018	2018-06-18	$H1_{av}N1$	1C.2.1	MT378971	MT378972	MT378973	MT378974	MT378975	MT378976	MT378977	MT378978	This study
A/swine/France/22-180323/2018	2018-06-28	$H1_{av}N1$	1C.2.1	MT378763	MT378764	MT378765	MT378766	MT378767	MT378768	MT378769	MT378770	This study
A/swine/France/59-180334/2018	2018-07-10	$H1_{av}N1$	1C.2.1 (C)	MT379283	MT379284	MT379285	MT379286	MT379287	MT379288	MT379289	MT379290	This study
A/swine/France/59-180359/2018	2018-08-10	$H1_{av}N1$	1C.2.1 (C)	MT379267	MT379268	MT379269	MT379270	MT379271	MT379272	MT379273	MT379274	This study
A/swine/France/56-180360/2018	2018-08-13	$H1_{av}N1$	1C.2.1	MT379155	MT379156	MT379157	MT379158	MT379159	MT379160	MT379161	MT379162	This study
A/swine/France/12-180371/2018	2018-09-03	$H1_{av}N1$	1C.2.1 (A)	MT378635	MT378636	MT378637	MT378638	MT378639	MT378640	MT378641	MT378642	This study
A/swine/France/56-180406/2018	2018-10-01	$H1_{hu}N2$	1B.1.2.3	MT379163	MT379164	MT379165	MT379166	MT379167	MT379168	MT379169	MT379170	This study
A/swine/France/49-180429/2018	2018-10-15	$H1_{av}N1$	1C.2.1 (A)	MT379027	MT379028	MT379029	MT379030	MT379031	MT379032	MT379033	MT379034	This study
A/swine/France/56-180424/2018	2018-10-23	$H1_{av}N2$	1C.2	MT379171	MT379172	MT379173	MT379174	MT379175	MT379176	MT379177	MT379178	This study
A/swine/France/35-180443/2018	2018-10-26	$H1_{hu}N2$	1B.1.2.3 (Δ146-147)	MT378979	MT378980	MT378981	MT378982	MT378983	MT378984	MT378985	MT378986	This study
A/swine/France/72-180446/2018	2018-10-29	$H1_{av}N1$	1C.2.1 (A)	MT379379	MT379380	MT379381	MT379382	MT379383	MT379384	MT379385	MT379386	This study
A/swine/France/22-180459/2018	2018-11-09	$H1_{hu}N2$	1B.1.2.3	MT378771	MT378772	MT378773	MT378774	MT378775	MT378776	MT378777	MT378778	This study
A/swine/France/53-180462/2018	2018-11-12	$H1_{av}N1$	1C.2.1 (A)	MT379099	MT379100	MT379101	MT379102	MT379103	MT379104	MT379105	MT379106	This study
A/swine/France/59-180465/2018	2018-11-13	$H1_{av}N1$	1C.2.1 (C)	MT379275	MT379276	MT379277	MT379278	MT379279	MT379280	MT379281	MT379282	This study
A/swine/France/12-180474/2018	2018-11-14	$H1_{av}N1$	1C.2.1 (A)	MT378643	MT378644	MT378645	MT378646	MT378647	MT378648	MT378649	MT378650	This study
A/swine/France/56-180472/2018	2018-11-16	$H1_{av}N1$	1C.2.1 (A)	MT379187	MT379188	MT379189	MT379190	MT379191	MT379192	MT379193	MT379194	This study
A/swine/france/56-180470/2018	2018-11-22	$H1_{hu}N2$	1B.1.2.3	MT379179	MT379180	MT379181	MT379182	MT379183	MT379184	MT379185	MT379186	This study

A/swine/France/16-180487/2018	2018-11-27	$H1_{av}N1$	1C.2.1 (A)	MT378651	MT378652	MT378653	MT378654	MT378655	MT378656	MT378657	MT378658	This study
A/swine/France/44-180495/2018	2018-11-29	$H1_{av}N1$	1C.2.1 (A)	MT379019	MT379020	MT379021	MT379022	MT379023	MT379024	MT379025	MT379026	This study
A/swine/France/35-190039/2018	2018-12-11	$H1_{av}N2$	1C.2.1 (A)	MT378987	MT378988	MT378989	MT378990	MT378991	MT378992	MT378993	MT378994	This study
A/swine/France/86-180497/2018	2018-12-13	$H1_{av}N1$	1C.2.1 (A)	MT379403	MT379404	MT379405	MT379406	MT379407	MT379408	MT379409	MT379410	This study

The asterisks indicates strains from clade HA-1B.1.2.3 harboring one (*) or two (**) amino acid deletions in position 146-147 in HA sequence but which did not clustered with strains of 1B.1.2.3 (Δ146-147) group.

References :

Chastagner A, Bonin E, Fablet C, Quéguiner S, Hirchaud E, Lucas P, Gorin S, Barbier N, Béven V, Garin E, Blanchard Y, Rose N, Hervé S, Simon G. 2019. Virus persistence in pig herds led to successive reassortment events between swine and human influenza A viruses, resulting in the emergence of a novel triple-reassortant swine influenza virus. Veterinary Research 50:77.

Dunham EJ, Dugan VG, Kaser EK, Perkins SE, Brown IH, Holmes EC, Taubenberger JK. 2009. Different evolutionary trajectories of European avian-like and classical swine H1N1 influenza A viruses. Journal of Virology 83:5485-94.

Franck N, Queguiner S, Gorin S, Eveno E, Fablet C, Madec F, Kuntz-Simon G. Molecular epidemiology of swine influenza virus in France: identifications of novel H1N1 reassortants, p 250. In (ed),

Krumbholz A, Lange J, Sauerbrei A, Groth M, Platzer M, Kanrai P, Pleschka S, Scholtissek C, Buttner M, Durrwald R, Zell R. 2014. Origin of the European avian-like swine influenza viruses. Journal of General Virology 95:2372-6.

Lycett SJ, Baillie G, Coulter E, Bhatt S, Kellam P, McCauley JW, Wood JL, Brown IH, Pybus OG, Leigh Brown AJ, Combating Swine Influenza Initiative CC. 2012. Estimating reassortment rates in co-circulating Eurasian swine influenza viruses. J Gen Virol 93:2326-36.

Marozin S, Gregory V, Cameron K, Bennett M, Valette M, Aymard M, Foni E, Barigazzi G, Lin Y, Hay A. 2002. Antigenic and genetic diversity among swine influenza A H1N1 and H1N2 viruses in Europe. Journal of General Virology 83:735-745.

Watson SJ, Langat P, Reid SM, Lam TT-Y, Cotten M, Kelly M, Van Reeth K, Qiu Y, Simon G, Bonin E, Foni E, Chiapponi C, Larsen L, Hjulsager C, Markowska-Daniel I, Urbaniak K, Dürrwald R, Schlegel M, Huovilainen A, Davidson I, Dán Á, Loeffen W, Edwards S, Bublot M, Vila T, Maldonado J, Valls L, Consortium E, Brown IH, Pybus OG, Kellam P. 2015. Molecular Epidemiology and Evolution of Influenza Viruses Circulating within European Swine between 2009 and 2013. Journal of Virology 89:9920-9931.
