# Supplementary material for: Genetic and Antigenic Evolution of European Swine Influenza A Viruses of HA-1C (Avian-Like) and HA-1B (Human-Like) Lineages in France from 2000 to 2018
Source: Viruses. 2020 Nov 13;12(11):1304. doi: 10.3390/v12111304 (PMC7697621; doi:10.3390/v12111304)
Supplement: Supplementary file 1 [file viruses-12-01304-s001.zip › Table_S2_Residues_involve_patho_virulence_fitness_Vf.pdf]

Table S2. List of residues identified as possibly involved in changing the pathogenicity, virulence or fitness of IAVs

Encoding protein – amino acid position	Effect described in literature	References
PB2-9	D9N increases virulence	[1, 2]
PB2-45	Impacts thermo-sensitive phenotype	[3]
PB2-158	E158K/G increase pathogenicity of H4N6 and H1N1pdm viruses	[4-6]
PB2-199	199S increases pathogenicity of H5N1 in mice	[2, 5, 7, 8]
PB2-251	R251K increases replication and pathogenicity	[9, 10]
PB2-253	D253N increases pathogenicity	[5, 11, 12]
PB2-265	N265S thermo-sensitive phenotype	[13-15]
PB2-271	T271A increases polymerase activity	[5, 7, 16]
PB2-339	K339T modulates polymerase activity and virulence	[17]
PB2-368	368K increases pathogenicity when combined with PB2-627K	[18]
PB2-431	M431I reduces Pimodivir susceptibility and virulence	[19, 20]
PB2-478	V478L thermo-sensitive phenotype	[14, 21]
PB2-504	Interacts with residue PA-550 in RNAP II degradation	[22]
PB2-588	T588I increases virulence	[7, 8, 23]
PB2-590	S590 compensates mutation PB2-E627K with PB2-R591	[5, 24, 25]
PB2-591	Q591R/K increases pathogenicity and compensates mutation PB2-E627K	[5, 24-26]
PB2-627	Major determinant of transcription / replication efficiency E627K increases pathogenicity of H4N6 but attenuates swine H3N2	[5, 6, 8, 27-30]
PB2-685	increases virulence when combined mutation in HA-190 or HA-212	[31]
PB2-701	D701N compensates effect of mutation PB2-E627K and enhances replication and pathogenicity of H1N1pdm and $H1_{av}N1$ viruses	[5, 9, 32, 33]
PB2-714	714R increases the cap-binding efficiency of PB2 when combined with PB2-701N	[5]
PB1-27	D27N increases polymerase activity	[34]
PB1-180	G180E enhances virus growth kinetics	[35]
PB1-181	I181T increases virulence of H5N2 in mice	[36]
PB1-216	S216G enhances virus growth kinetics and virulence	[35, 37]
PB1-229	K229R reduces favipiravir susceptibility and viral fitness	[19]
PB1-265	K265N attenuates virus	[14]
PB1-353	K353R impacts virulence	[38]
PB1-361	S361R enhances virus growth kinetics	[35]
PB1-391	K391E increases virus temperature sensitivity	[13-15]
PB1-456	H456Y plays a role in virulence and adaptation in mice	[39]
PB1-469	A469T enhances replication, pathogenicity and transmission	[40]
PB1-473	V473 increases polymerase activity, L473 decreases polymerase activity in H1N1pdm virus	[5, 41]
PB1-566	T566A could impact virulence	[38]

PB1-581	E581G increases virus temperature sensitivity	[13, 15]
PB1-591	V591I attenuates virus	[14]
PB1-598	Increase polymerase activity	[5, 41]
PB1-621	Q621R could enhance virus growth kinetics	[35]
PB1-653	P653L increases polymerase activity	[19]
PB1-654	N654S could enhance virus growth kinetics	[35]
PB1-661	A661T increases virus temperature sensitivity	[13-15]
PB1-F2-51	T51M decreases lethality	[42]
PB1-F2-56	V56A decreases lethality	[42]
PB1-F2-62	L62: impacts inflammatory activity and enhanced pathogenicity of bacterial superinfection /P62: impacts antimicrobial activity	[42-44]
PB1-F2-66	N66S increases virulence in mice with limited effect in H1N1pdm virus<br>66S enhances pathogenicity of bacterial superinfection	[44-46]
PB1-F2-68	68I : responsible for promotion of the peptide’s cytotoxicity and permeabilization of the mitochondrial membrane (in combination with PB1-F2-69L and -70V)	[47]
PB1-F2-69	69L: responsible for promotion of the peptide’s cytotoxicity and permeabilization of the mitochondrial membrane (in combination with PB1-F2-68I and -70V)	[47]
PB1-F2-70	70V: responsible for promotion of the peptide’s cytotoxicity and permeabilization of the mitochondrial membrane (in combination with PB1-F2-68I and -69L)	[47]
PB1-F2-79	R79: impacts inflammatory activity and enhances pathogenicity of bacterial superinfection /Q79: impacts antimicrobial activity	[7, 42-44]
PB1-F2-82	L82: Impacts inflammatory activity and enhances pathogenicity of bacterial superinfection /S82: impacts antimicrobial activity	[7, 42-44]
PB1-F2-87	E87G decreases lethality	[7]
PA-85	85I enhances activity of the Cal polymerase	[48]
PA-129	I129T enhances replication, pathogenicity and transmission	[40]
PA-142	142N increases pathogenicity of H5N1 in mice, 142E affects pathogenicity when combined with PB2-627K	[2, 21]
PA-224	Affects endonuclease activity<br>S224P highly virulent phenotype of H5N1 in duck	[49, 50]
PA-295	L295P is responsible for transcription and replication activity	[51]
PA-298	E298K contributes to virulence	[52]
PA-336	336M increases avian polymerase activity in mammals cells	[5, 48]
PA-343	A343T increases H1N1pdm virulence	[8, 38]
PA-383	N383D highly virulent phenotype of H5N1 in duck	[21, 50]
PA-409	S409N increases transmissibility of H1N1pdm virus	[7, 8]
PA-421	421I increases pathogenicity of H5N1 in mice	[2, 18, 21]
PA-552	552S increases polymerase activity	[5, 7, 8, 53]
PA-615	615N increases polymerase activity	[5]
PA-666	L666F reduces polymerase activity	[19]
PA-713	H713R defects packaging	[54]
PA-714	A714G defects packaging	[54]
NP-16	G16D increases MxA resistance	[7, 8, 55]

NP-34	34G increases temperature sensitivity	[13]
NP-41	41V enhances polymerase activities and replication of H7N9	[56]
NP-48	affectes MxA resistance	[57]
NP-53	53D increases MxA resistance	[55]
NP-64	D64G increases thermosensitivity of strains	[13]
NP-72	D72A affects incorporation of vRNAs into VLPs	[58]
NP-74	R74A affects incorporation of vRNAs into VLPs	[58]
NP-98	affects MxA resistance	[21, 57]
NP-99	affects MxA resistance	[57]
NP-100	100I/V increases MxA resistance	[7, 8, 55]
NP-101	D101G contributes to virulence	[28, 51]
NP-108	Thermos-sensitive phenotype	[3]
NP-109	I109T increases replication in chicken	[7, 8, 59]
NP-113	K113A affects incorporation of vRNAs into VLPs	[58]
NP-150	R150A affects viral-genome replication and transcription	[58]
NP-156	R156A affects incorporation of vRNAs into VLPs	[58]
NP-174	R174A affects incorporation of vRNAs into VLPs	[58]
NP-175	R175A affects incorporation of vRNAs into VLPs	[58]
NP-188	T188 regulates viral replication by controlling NES2-dependent NP nuclear export and the polymerase activity of the vRNP complex	[60]
NP-195	R195A affects incorporation of vRNAs into VLPs	[60]
NP-199	R199A affects incorporation of vRNAs into VLPs	[60]
NP-204	S204 requires for NP binding to viral polymerase	[61]
NP-207	W207 requires for NP binding to viral polymerase	[61]
NP-208	R208A affects viral-genome replication and transcription	[58, 61]
NP-210	210D enhances polymerase activities and replication of H7N9	[56]
NP-213	R213A affects viral-genome replication and transcription	[58]
NP-214	Involves in the conversion of filamentous virions into spherical virions	[7, 8, 62]
NP-217	Involves in the conversion of filamentous virions into spherical virions	[21, 62, 63]
NP-239	M239L thermos-sensistive lesion	[64]
NP-253	Involves in the conversion of filamentous virions into spherical virions	[62]
NP-254	E254A affects viral-genome replication and transcription	[58]
NP-260	A260R affects viral-genome replication and transcription	[58]
NP-273	K273A affects viral-genome replication and transcription	[58]
NP-283	L283P increases MxA resistance	[7, 8, 55]
NP-289	289H increases MxA resistance	[55]
NP-305	305K increases MxA resistance	[7, 8, 28, 55]
NP-313	F313Y/V increases MxA resistance	[7, 8, 55]
NP-316	316M increases MxA resistance	[55]
NP-319	N319K enhances viral replication	[5]
NP-325	K325A affects incorporation of vRNAs into VLPs	[58]
NP-337	A337R affects viral-genome replication and transcription	[58]
NP-343	NP-V343L mutation switched from being highly BNP-sensitive to moderately BNP-resistant	[65]
NP-350	350K increases MxA resistance	[55]

NP-351	351K increases MxA resistance	[55]
NP-353	353I/S increases MxA resistance	[21, 28, 55]
NP-355	R355A affects viral-genome replication and transcription	[58]
NP-357	Q357K increases MxA resistance and virulence affects also the pathogenicity when associated to PB2-627K	[7, 8, 55, 66]
NP-361	R361A affects incorporation of vRNAs into VLPs	[58]
NP-387	A387R affects viral-genome replication and transcription	[58]
NP-405	Q405A affects viral-genome replication and/or transcription	[58]
NP-412	F412A affects viral-genome replication and/or transcription	[58]
NP-488	F488A affects viral-genome replication and/or transcription	[58]
NP-489	F489A affects viral-genome replication and/or transcription	[58]
M1-30	Influences the morphology of virions	[62]
M1-41	P41A alters virion morphology, reducing the number and length of filamentous virions, as well as reducing the neuraminidase activity of virions	[67]
M1-90	P90 inhibes ribonucleoproteins transcription	[68]
M1-95	R95K described in pathogenic strains of H5N1	[69]
M1-101	R101S affects viral replication and thermo-sensibility	[70]
M1-105	R105S affects viral replication and thermo-sensibility	[70]
M1-108	T108 inhibes ribonucleoproteins transcription	[68]
M1-132	Role in nuclear import	[71]
M1-142	Impacts morphology of virions	[72]
M1-207	Impacts morphology of virions	[72]
M1-209	Impacts morphology of virions	[72]
M1-224	N224S described in pathogenic strains of H5N1	[69]
M1-230	R230K described in pathogenic strains of H5N1	[69]
M2-55	C55F increases transmissibility	[18]
NS1-18	V18A thermo-sensitive phenotype	[73]
NS1-35	R35 and R46 critical for binding to and blocking activation of PKR and for efficient virus propagation	[74]
NS1-38	critical role in RNA binding and inhibition of IFN production	[75]
NS1-42	P42S increases virulence in mice<br>S42D attenuates virulence	[76-78]
NS1-44	R44K thermo-sensitive phenotype	[73]
NS1-46	R35 and R46 critical for binding to and blocking activation of PKR and for efficient virus propagation	[74]
NS1-48	S48A/D affects virus replication	[78]
NS1-55	K55E, K66E, and C133F restored ability to conteract interferon response	[79]
NS1-64	I64T affects interferon response and virulence	[80]
NS1-66	K55E, K66E, and C133F restored ability to conteract interferon response	[79]
NS1-92	E92 increases clinical signs	[8]
NS1-97	E97A decreases TRIM25 activity	[81]
NS1-103	F103L increases virulence<br>F103 critical for CPSF binding	[82-84]
NS1-106	M106I increases virulence<br>M106 critical for CPSF binding	[82-84]
NS1-123	Suspected to have a role in human adaptation, and virulence	[49, 85]

NS1-133	K55E, K66E, and C133F restore ability to conteract interferon response	[79]
NS1-149	A149 antagonizes interferon production and increases pathogenicity of H5N1 in chicken	[86]
NS1-171	A171Y increases NS1 expression and reduces IFN expression	[87, 88]
NS1-195	S195P thermo-sensitive phenotype	[73]
NS1-200	N200S increase virulence of H5N1 in ferret, enhanced IFN 1 activity	[89]
NS1-205	N205K enhanced replication, pathogenicity and transmission G205R increase virulence of H5N1 in ferret, enhanced IFN 1 activity	[40, 89]
NS1-215	T215A attenuates viral replication	[18, 28, 78]
NEP-47	T47A increases virulence of H5N1 in ferret	[89]
NEP-48	T48N enhances replication, pathogenicity and transmission	[40]
NEP-51	M51I increases virulence of H5N1 in ferret	[89]
NEP-69	E69G increases virulence H5N2 in mice	[36]

References

1. Graef, K.M., et al., *The PB2 subunit of the influenza virus RNA polymerase affects virulence by interacting with the mitochondrial antiviral signaling protein and inhibiting expression of beta interferon.* Journal of Virology 2010. **84**(17): p. 8433-45.
2. Kim, J.H., et al., *Role of host-specific amino acids in the pathogenicity of avian H5N1 influenza viruses in mice.* Journal of General Virology, 2010. **91**(Pt 5): p. 1284-9.
3. Kiseleva, I.V., et al., *PB2 and PA genes control the expression of the temperature-sensitive phenotype of cold-adapted B/USSR/60/69 influenza master donor virus.* Journal of General Virology, 2010. **91**(Pt 4): p. 931-7.
4. Zhou, B., et al., *PB2 residue 158 is a pathogenic determinant of pandemic H1N1 and H5 influenza a viruses in mice.* Journal of Virology 2011. **85**(1): p. 357-65.
5. Manz, B., M. Schwemmle, and L. Brunotte, *Adaptation of avian influenza A virus polymerase in mammals to overcome the host species barrier.* J Virol, 2013. **87**(13): p. 7200-9.
6. Xu, G., et al., *Mutations in PB2 and HA enhanced pathogenicity of H4N6 avian influenza virus in mice.* Journal of General Virology, 2019.
7. Pan, C., et al., *Genomic signature and mutation trend analysis of pandemic (H1N1) 2009 influenza A virus.* PLoS One, 2010. **5**(3): p. e9549.
8. Makkoch, J., et al., *Whole genome characterization, phylogenetic and genome signature analysis of human pandemic H1N1 virus in Thailand, 2009-2012.* PLoS One, 2012. **7**(12): p. e51275.
9. Dunham, E.J., et al., *Different evolutionary trajectories of European avian-like and classical swine H1N1 influenza A viruses.* Journal of Virology 2009. **83**(11): p. 5485-94.
10. Cai, M., et al., *The R251K Substitution in Viral Protein PB2 Increases Viral Replication and Pathogenicity of Eurasian Avian-like H1N1 Swine Influenza Viruses.* Viruses, 2020. **12**(1).
11. Mok, C.K., et al., *Amino Acid Residues 253 and 591 of the PB2 Protein of Avian Influenza Virus A H9N2 Contribute to Mammalian Pathogenesis.* Journal of Virology 2011. **85**(18): p. 9641-5.

12. Zhang, J., et al., *The D253N Mutation in the Polymerase Basic 2 Gene in Avian Influenza (H9N2) Virus Contributes to the Pathogenesis of the Virus in Mammalian Hosts.* Virol Sin, 2018. **33**(6): p. 531-537.
13. Jin, H., et al., *Multiple amino acid residues confer temperature sensitivity to human influenza virus vaccine strains (FluMist) derived from cold-adapted A/Ann Arbor/6/60.* Virology, 2003. **306**(1): p. 18-24.
14. He, W., et al., *Molecular basis of live-attenuated influenza virus.* PLoS One, 2013. **8**(3): p. e60413.
15. Broadbent, A.J., et al., *The temperature-sensitive and attenuation phenotypes conferred by mutations in the influenza virus PB2, PB1, and NP genes are influenced by the species of origin of the PB2 gene in reassortant viruses derived from influenza A/California/07/2009 and A/WSN/33 viruses.* J Virol, 2014. **88**(21): p. 12339-47.
16. Bussey, K.A., et al., *PB2 residue 271 plays a key role in enhanced polymerase activity of influenza A viruses in mammalian host cells.* Journal of Virology 2010. **84**(9): p. 4395-406.
17. Liu, Y., et al., *Structural and functional characterization of K339T substitution identified in the PB2 subunit cap-binding pocket of influenza A virus.* J Biol Chem, 2013. **288**(16): p. 11013-23.
18. Miotto, O., et al., *Complete-proteome mapping of human influenza a adaptive mutations: implications for human transmissibility of zoonotic strains.* PLoS One, 2010. **5**(2): p. e9025.
19. Mifsud, E.J., F.G. Hayden, and A.C. Hurt, *Antivirals targeting the polymerase complex of influenza viruses.* Antiviral Research, 2019. **169**: p. 104545.
20. Xu, C., et al., *A single amino acid at position 431 of the PB2 protein determines the virulence of H1N1 swine influenza viruses in mice.* Journal of Virology, 2020 in press: p. JVI.01930-19.
21. Lindstrom, S.E., N.J. Cox, and A. Klimov, *Genetic analysis of human H2N2 and early H3N2 influenza viruses, 1957-1972: evidence for genetic divergence and multiple reassortment events.* Virology, 2004. **328**(1): p. 101-19.
22. Llompart, C.M., A. Nieto, and A. Rodriguez-Frandsen, *Specific residues of PB2 and PA influenza virus polymerase subunits confer the ability for RNA polymerase II degradation and virus pathogenicity in mice.* J Virol, 2014.
23. Zhao, Z., et al., *PB2-588I enhances 2009 H1N1 pandemic influenza virus virulence by increasing viral replication and exacerbating PB2 inhibition of beta interferon expression.* Journal of Virology 2014. **88**(4): p. 2260-7.
24. Mehle, A. and J.A. Doudna, *Adaptive strategies of the influenza virus polymerase for replication in humans.* PNAS - Proceedings of the National Academy of Sciences of the United States of America, 2009. **106**(50): p. 21312-6.
25. Liu, Q., et al., *Combination of PB2 271A and SR polymorphism at positions 590/591 is critical for viral replication and virulence of swine influenza virus in cultured cells and in vivo.* J Virol, 2012. **86**(2): p. 1233-7.
26. Yamada, S., et al., *Biological and structural characterization of a host-adapting amino acid in influenza virus.* PLoS Pathogens, 2010. **6**(8): p. e1001034.
27. Subbarao, K., W. London, and B.R. Murphy, *A Single Amino Acid in the PB2 Gene of Influenza A Virus Is a Determinant of Host Range.* Journal Of Virology, 1993. **67**(4): p. 1761-1764.
28. Tamuri, A.U., et al., *Identifying changes in selective constraints: host shifts in influenza.* PLoS Comput Biol, 2009. **5**(11): p. e1000564.
29. Gong, X.Q., et al., *The PB2-K627E mutation attenuates H3N2 swine influenza virus in cultured cells and in mice.* Res Vet Sci, 2018. **117**: p. 54-56.
30. Nilsson, B.E., A.J. Te Velthuis, and E. Fodor, *Role of the PB2 627 Domain in Influenza A Virus Polymerase Function.* Journal of Virology 2017. **91**(7).
31. Yang, W., et al., *Increased virulence of a PB2/HA mutant of an avian H9N2 influenza strain after three passages in porcine differentiated airway epithelial cells.* Veterinary Microbiology, 2017. **211**(Supplement C): p. 129-134.
32. Zhou, B., et al., *Asparagine Substitution at PB2 Residue 701 Enhances the Replication, Pathogenicity, and Transmission of the 2009 Pandemic H1N1 Influenza A Virus.* PLoS One, 2013. **8**(6): p. e67616.

33. Liu, S., et al., *Substitution of D701N in the PB2 protein could enhance the viral replication and pathogenicity of Eurasian avian-like H1N1 swine influenza viruses.* Emerg Microbes Infect, 2018. **7**(1): p. 75.
34. Binh, N.T., et al., *Involvement of the N-terminal portion of influenza virus RNA polymerase subunit PB1 in nucleotide recognition.* Biochem Biophys Res Commun, 2013.
35. Plant, E.P., et al., *Mutations to A/Puerto Rico/8/34 PB1 gene improves seasonal reassortant influenza A virus growth kinetics.* Vaccine, 2012. **31**(1): p. 207-12.
36. Wu, H., et al., *Amino acid substitutions involved in the adaptation of a novel highly pathogenic H5N2 avian influenza virus in mice.* Virology Journal, 2016. **13**(1).
37. Lin, R.-W., et al., *Naturally occurring mutations in PB1 affect influenza A virus replication fidelity, virulence, and adaptability.* Journal of biomedical science, 2019. **26**(1): p. 55-55.
38. Xu, L., et al., *Genomic polymorphism of the pandemic A (H1N1) influenza viruses correlates with viral replication, virulence, and pathogenicity in vitro and in vivo.* PLoS One, 2011. **6**(6): p. e20698.
39. Hiromoto, Y., et al., *Characterization of low virulent strains of highly pathogenic A/Hong Kong/156/97 (H5N1) virus in mice after passage in embryonated hens' eggs.* Virology, 2000. **272**(2): p. 429-37.
40. Wei, K., et al., *Influenza a virus acquires enhanced pathogenicity and transmissibility after serial passages in Swine.* Journal of Virology 2014. **88**(20): p. 11981-94.
41. Xu, C., et al., *Amino acids 473V and 598P of PB1 from an avian-origin influenza A virus contribute to polymerase activity, especially in mammalian cells.* Journal of General Virology, 2012. **93**(Pt 3): p. 531-40.
42. Kosik, I., J. Holly, and G. Russ, *PB1-F2 expedition from the whole protein through the domain to aa residue function.* Acta Virol, 2013. **57**(2): p. 138-48.
43. Alymova, I.V., et al., *Immunopathogenic and antibacterial effects of H3N2 influenza A virus PB1-F2 map to amino acid residues 62, 75, 79, and 82.* Journal of Virology 2011. **85**(23): p. 12324-33.
44. Weeks-Gorospe, J.N., et al., *Naturally Occurring Swine Influenza A Virus PB1-F2 Phenotypes That Contribute to Superinfection with Gram-Positive Respiratory Pathogens.* Journal of Virology, 2012. **86**(17): p. 9035-9043.
45. Hai, R., et al., *PB1-F2 expression by the 2009 pandemic H1N1 influenza virus has minimal impact on virulence in animal models.* Journal of Virology 2010. **84**(9): p. 4442-50.
46. Ozawa, M., et al., *Impact of amino acid mutations in PB2, PB1-F2, and NS1 on the replication and pathogenicity of pandemic (H1N1) 2009 influenza viruses.* Journal of Virology 2011. **85**(9): p. 4596-601.
47. Alymova, I.V., et al., *A novel cytotoxic sequence contributes to influenza A protein PB1-F2 pathogenicity and predisposition to secondary bacterial infection.* Journal of Virology 2013.
48. Bussey, K.A., et al., *PA residues in the 2009 H1N1 pandemic influenza virus enhance avian influenza virus polymerase activity in mammalian cells.* Journal of Virology 2011. **85**(14): p. 7020-8.
49. Giria, M.T., et al., *Genomic signatures and antiviral drug susceptibility profile of A(H1N1)pdm09.* Journal of Clinical Virology, 2012. **53**(2): p. 140-4.
50. Song, J., et al., *The PA protein directly contributes to the virulence of H5N1 avian influenza viruses in domestic ducks.* Journal of Virology 2011. **85**(5): p. 2180-8.
51. Ilyushina, N.A., et al., *Adaptation of pandemic H1N1 influenza viruses in mice.* J Virol, 2010. **84**(17): p. 8607-16.
52. Ye, J., et al., *Variations in the hemagglutinin of the 2009 H1N1 pandemic virus: potential for strains with altered virulence phenotype?* PLoS Pathogens, 2010. **6**(10): p. e1001145.
53. Mehle, A., et al., *Reassortment and Mutation of the Avian Influenza Virus Polymerase PA Subunit Overcome Species Barriers.* Journal of Virology, 2012. **86**(3): p. 1750-1757.
54. Liang, Y., et al., *Mutational analyses of packaging signals in influenza virus PA, PB1, and PB2 genomic RNA segments.* J Virol, 2008. **82**(1): p. 229-36.
55. Manz, B., et al., *Pandemic influenza A viruses escape from restriction by human MxA through adaptive mutations in the nucleoprotein.* PLoS Pathog, 2013. **9**(3): p. e1003279.

56. Zhu, W., et al., *Residues 41V and/or 210D in the NP protein enhance polymerase activities and potential replication of novel influenza (H7N9) viruses at low temperature.* Virology Journal, 2015. **12**: p. 71.
57. Dornfeld, D., et al., *Eurasian avian-like swine influenza A viruses escape human MxA restriction by distinct mutations in their nucleoprotein.* J Virol, 2018.
58. Li, Z., et al., *Mutational analysis of conserved amino acids in the influenza a virus nucleoprotein.* Journal of Virology 2009. **83**(9): p. 4153-62.
59. Tada, T., et al., *Emergence of Avian Influenza Viruses with Enhanced Transcription Activity by a Single Amino Acid Substitution in the Nucleoprotein during Replication in Chicken Brains.* Journal of Virology 2011. **85**(19): p. 10354-63.
60. Li, Y., et al., *Phosphorylation and dephosphorylation of threonine 188 in nucleoprotein is crucial for the replication of influenza A virus.* Virology, 2018. **520**: p. 30-38.
61. Marklund, J.K., et al., *Sequence in the Influenza A Virus Nucleoprotein Required for Viral Polymerase Binding and RNA Synthesis.* Journal of Virology, 2012. **86**(13): p. 7292-7297.
62. Bialas, K.M., et al., *Specific nucleoprotein residues affect influenza virus morphology.* Journal of Virology 2013.
63. de la Rosa-Zamboni, D., et al., *Molecular characterization of the predominant influenza A(H1N1)pdm09 virus in Mexico, December 2011-February 2012.* PLoS One, 2012. **7**(11): p. e50116.
64. Noton, S.L., et al., *Studies of an influenza A virus temperature-sensitive mutant identify a late role for NP in the formation of infectious virions.* Journal of Virology 2009. **83**(2): p. 562-71.
65. Narkpuk, J., et al., *Single nucleoprotein residue determines influenza A virus sensitivity to an intertypic suppression mechanism.* Virology, 2017. **506**: p. 99-109.
66. Zhu, W., et al., *Mammalian-adaptive mutation NP-Q357K in Eurasian H1N1 Swine Influenza viruses determines the virulence phenotype in mice.* Emerg Microbes Infect, 2019. **8**(1): p. 989-999.
67. Campbell, P.J., et al., *Residue 41 of the Eurasian Avian-Like Swine Influenza A Virus Matrix Protein Modulates Virion Filament Length and Efficiency of Contact Transmission.* Journal of Virology 2014. **88**(13): p. 7569-7577.
68. Ye, Z.P., N.W. Baylor, and R.R. Wagner, *Transcription-inhibition and RNA-binding domains of influenza A virus matrix protein mapped with anti-idiotypic antibodies and synthetic peptides.* Journal of Virology 1989. **63**(9): p. 3586-94.
69. Liu, N., et al., *Identification of amino acid substitutions in avian influenza virus (H5N1) matrix protein 1 by using nanoelectrospray MS and MS/MS.* J Am Soc Mass Spectrom, 2009. **20**(2): p. 312-20.
70. Xie, H., et al., *The compensatory G88R change is essential in restoring the normal functions of influenza A/WSN/33 virus matrix protein 1 with a disrupted nuclear localization signal.* J Virol, 2013. **87**(1): p. 345-53.
71. Wang, S., et al., *Tyrosine 132 phosphorylation of influenza A virus M1 protein is crucial for virus replication by controlling the nuclear import of M1.* Journal of Virology 2013. **87**(11): p. 6182-91.
72. Bialas, K.M., E.A. Desmet, and T. Takimoto, *Specific residues in the 2009 H1N1 swine-origin influenza matrix protein influence virion morphology and efficiency of viral spread in vitro.* PLoS One, 2012. **7**(11): p. e50595.
73. Garaigorta, U., A.M. Falcon, and J. Ortin, *Genetic Analysis of Influenza Virus NS1 Gene: a Temperature-Sensitive Mutant Shows Defective Formation of Virus Particles.* J Virol, 2005. **79**(24): p. 15246-57.
74. Schierhorn, K.L., et al., *Influenza A Virus Virulence Depends on Two Amino Acids in the N-Terminal Domain of Its NS1 Protein To Facilitate Inhibition of the RNA-Dependent Protein Kinase PKR.* Journal of Virology 2017. **91**(10).
75. Donelan, N.R., C.F. Basler, and A. Garcia-Sastre, *A recombinant influenza A virus expressing an RNA-binding-defective NS1 protein induces high levels of beta interferon and is attenuated in mice.* Journal of Virology 2003. **77**(24): p. 13257-66.

76. Jiao, P., et al., *A single-amino-acid substitution in the NS1 protein changes the pathogenicity of H5N1 avian influenza viruses in mice.* Journal of Virology 2008. **82**(3): p. 1146-54.
77. Cheng, J., et al., *Effects of the S42 residue of the H1N1 swine influenza virus NS1 protein on interferon responses and virus replication.* Virol J, 2018. **15**(1): p. 57.
78. Hsiang, T.-Y., L. Zhou, and R.M. Krug, *Roles of the Phosphorylation of Specific Serines and Threonines in the NS1 Protein of Human Influenza A Viruses.* Journal of Virology, 2012. **86**(19): p. 10370-10376.
79. Li, J., et al., *Three amino acid substitutions in the NS1 protein change the virus replication of H5N1 influenza virus in human cells.* Virology, 2018. **519**: p. 64-73.
80. DeDiego, M.L., et al., *NS1 Protein Mutation I64T Affects Interferon Responses and Virulence of Circulating H3N2 Human Influenza A Viruses.* Journal of Virology 2016. **90**(21): p. 9693-9711.
81. Goldstein, T., et al., *Pandemic H1N1 influenza isolated from free-ranging Northern Elephant Seals in 2010 off the central California coast.* PLoS One, 2013. **8**(5): p. e62259.
82. Dankar, S., et al., *Influenza A virus NS1 gene mutations F103L and M106I increase replication and virulence.* Virology Journal, 2011. **8**(1): p. 13.
83. Dankar, S.K., et al., *Influenza A/Hong Kong/156/1997(H5N1) virus NS1 gene mutations F103L and M106I both increase IFN antagonism, virulence and cytoplasmic localization but differ in binding to RIG-I and CPSF30.* Virology Journal, 2013. **10**: p. 243.
84. Kochs, G., A. Garcia-Sastre, and L. Martinez-Sobrido, *Multiple anti-interferon actions of the influenza A virus NS1 protein.* J Virol, 2007. **81**(13): p. 7011-21.
85. Wang, C., et al., *Evolutionary characterization of the pandemic H1N1/2009 influenza virus in humans based on non-structural genes.* PLoS One, 2013. **8**(2): p. e56201.
86. Li, Z.J., et al., *The NSI gene contributes to the virulence of H5N1 avian influenza viruses.* Journal of Virology, 2006. **80**(22): p. 11115-11123.
87. Xu, J., et al., *Molecular phylogeny and evolutionary dynamics of influenza A nonstructural (NS) gene.* Infection, Genetics and Evolution, 2013.
88. Plant, E.P., et al., *Influenza virus NS1 protein mutations at position 171 impact innate interferon responses by respiratory epithelial cells.* Virus Res, 2017. **240**: p. 81-86.
89. Imai, H., et al., *The HA and NS genes of human H5N1 influenza A virus contribute to high virulence in ferrets.* PLoS Pathogens, 2010. **6**(9): p. e1001106.
