# Supplementary material for: Genetic and Antigenic Evolution of European Swine Influenza A Viruses of HA-1C (Avian-Like) and HA-1B (Human-Like) Lineages in France from 2000 to 2018
Source: Viruses. 2020 Nov 13;12(11):1304. doi: 10.3390/v12111304 (PMC7697621; doi:10.3390/v12111304)
Supplement: Supplementary file 1 [file viruses-12-01304-s001.zip › Table_S4_Specific_mutation_pattern_Vf.pdf]

Table S4. Specific mutation patterns identified with AVANA software which characterize deduced amino acid sequences of internal proteins from strains of the three genogroups A, B and C within HA-1C.2.1 clade

Mutual Information measures the dependence between two variables, i.e. the relationship between the amino acid residues observed at a site and the set of sequences in which they are observed, knowing their occurrence frequency in the other set of sequences.

* indicates virulence markers described in the literature, and the rows are colored in green or in red when the exact mutation has been proven to decrease or increase virulence, respectively.

(A) Specific mutation patterns in internal proteins of strains from HA-1C.2.1 clade group A

Amino acid (aa) position	Major aa (frequency) within HA-1C.2.1 strains of group A (n = 28)	Major aa (frequency) within HA-1C strains exclude group A (n = 67)	Mutual Information
PB1-F2-35	S (0.893)	L (0.841)	0.442
PB1-F2-37	L (0.821)	RQ (0.826)	0.571
PB1-F2-42	S (0.786)	Y (0.58)	0.522
PB1-F2-65	K (0.893)	R (0.812)	0.408
PB1-F2-87*	G (0.857)	E (0.87)	0.430
PA-388	N (0.857)	SG (0.913)	0.518
M2-88	N (0.714)	D (0.971)	0.521
NS1-66	ND (0.964)	K (0.725)	0.492
NS1-76	E (0.929)	A (0.841)	0.717
NS1-86	V (0.964)	A (0.797)	0.578

(B) Specific mutation patterns in internal proteins of strains from HA-1C.2.1 clade group B

Amino acid (aa) position	Major aa (frequency) within HA-1C.2.1 strains of group B (n = 8)	Major aa (frequency) within HA-1C strains exclude group B (n = 96)	Mutual Information
PB2-73	K (1)	Q (1)	1
PB2-82	T (1)	NS (1)	1
PB2-463	V (1)	I (0.989)	0.984
PB2-575	V (0.875)	MI (0.978)	0.691
PB1-364	I (0.875)	L (0.966)	0.639
PB1-619	N (1)	D (1)	1
PB1-F2-6	G (1)	D (0.966)	0.920
PB1-F2-29	R (1)	K (0.989)	0.975
PB1-F2-33	P (1)	L (0.876)	0.833
PB1-F2-37	Q (1)	RL (0.82)	0.771
PB1-F2-42	C (1)	YSF (0.989)	0.982
PB1-F2-46	T (0.875)	M (1)	0.717
PB1-F2-55	I (0.875)	T (0.888)	0.516
PB1-F2-57	F (1)	SY (0.978)	0.958
PB1-F2-66*	S (1)	N (0.978)	0.975
PA-66	G (1)	SN (0.978)	0.952
PA-204	K (1)	R (0.978)	0.952
PA-296	N (0.875)	S (1)	0.717
PA-330	V (1)	I (0.966)	0.958
PA-615*	R (1)	K (0.876)	0.767

PA-684	E (1)	G (0.966)	0.922
PA-716	R (1)	K (0.932)	0.824
PA-X-66	G (1)	SN (0.978)	0.952
PA-X-204	S (1)	GD (0.978)	0.952
PA-X-208	K (1)	R (0.933)	0.855
NP-377	I (1)	V (0.798)	0.636
NP-450	N (1)	S (0.933)	0.858
NS1-24	D (1)	N (0.876)	0.802
NS1-44	K (1)	R (0.899)	0.785
NS1-48*	I (1)	SN (1)	1
NS1-53	K (1)	E (1)	1
NS1-62	ND (1)	KR (0.978)	1
NS1-79	L (1)	ITM (0.966)	0.942
NS1-92*	E (1)	D (0.978)	0.932
NS1-171*	G (1)	D (0.944)	0.909
NS1-182	V (1)	I (0.966)	0.929
NS1-202	T (1)	A (0.899)	0.826
NS1-209	D (1)	NK (0.989)	0.977
NS1-214	L (1)	FY (0.944)	0.859
NS1-216	T (1)	PS (0.966)	0.930
NEP-14	V (1)	MT (0.944)	0.909

(C) Specific mutation patterns in internal proteins of strains from HA-1C.2.1 clade group C

Amino acid (aa) position	Major aa (frequency) within HA-1C.2.1 strains of group C (n = 9)	Major aa (frequency) within HA-1C strains exclude group C (n = 95)	Mutual Information
PB2-292	M (0.889)	I (0.75)	0.542
PB2-353	R (0.889)	K (1)	0.739
PB2-507	P (0.889)	Q (1)	0.739
PB2-718	R (0.889)	K (0.955)	0.649
PB1-52	K (0.889)	R (0.989)	0.718
PB1-257	A (0.889)	T (0.943)	0.638
PB1-264	DN (0.889)	E (1)	0.739
PB1-386	K (1)	R (0.977)	0.940
PB1-584	Q (0.889)	H (0.977)	0.741
PB1-654*	N (0.889)	S (0.977)	0.688
PB1-F2-8	L (0.778)	P (0.909)	0.465
PB1-F2-11	Q (0.889)	R (0.92)	0.606
PB1-F2-16	T (1)	I (0.966)	0.917
PB1-F2-21	K (0.889)	E (0.898)	0.907
PB1-F2-59	K (0.889)	R (0.955)	0.650
PB1-F2-63	F (0.889)	S (0.955)	0.653
PB1-F2-73	R (0.889)	K (1)	0.739
PA-13	V (0.889)	I (0.977)	0.684
PA-343*	A (1)	T (0.852)	0.772
PA-400	VNS (1)	I (0.875)	0.900
PA-X-13	V (0.889)	I (0.977)	0.684
PA-X-618	K (1)	Q (1)	1
PA-X-624	I (1)	V (0.943)	0.900

PA-X-638	I (1)	V (0.807)	0.643
M2-14	E (0.778)	G (0.92)	0.431
NS1-18	V (0.889)	I (0.966)	0.647
NS1-214	Y (0.667)	F (0.875)	0.619
NS1-217	ER (0.889)	K (0.989)	0.725
NEP-57	TY (0.889)	SF (0.966)	0.710
